# Shape adaptable and highly resilient 3D braided triboelectric nanogenerators as e-textiles for power and sensing

**DOI:** 10.1038/s41467-020-16642-6

**Published:** 2020-06-08

**Authors:** Kai Dong, Xiao Peng, Jie An, Aurelia Chi Wang, Jianjun Luo, Baozhong Sun, Jie Wang, Zhong Lin Wang

**Affiliations:** 1 Beijing Institute of Nanoenergy and Nanosystems, Chinese Academy of Sciences, Beijing, 100083 P. R. China; 2 College of Nanoscience and Technology, University of Chinese Academy of Sciences, Beijing, 100049 P. R. China; 3 School of Material Science and Engineering, Georgia Institute of Technology, Atlanta, GA 30332-0245 USA; 4 College of Textiles, Donghua University, Shanghai, 201020 P. R. China

**Keywords:** Devices for energy harvesting, Electronic devices, Sensors

## Abstract

Combining traditional textiles with triboelectric nanogenerators (TENGs) gives birth to self-powered electronic textiles (e-textiles). However, there are two bottlenecks in their widespread application, low power output and poor sensing capability. Herein, by means of the three-dimensional five-directional braided (3DB) structure, a TENG-based e-textile with the features of high flexibility, shape adaptability, structural integrity, cyclic washability, and superior mechanical stability, is designed for power and sensing. Due to the spatial frame-column structure formed between the outer braided yarn and inner axial yarn, the 3DB-TENG is also endowed with high compression resilience, enhanced power output, improved pressure sensitivity, and vibrational energy harvesting ability, which can power miniature wearable electronics and respond to tiny weight variations. Furthermore, an intelligent shoe and an identity recognition carpet are demonstrated to verify its performance. This study hopes to provide a new design concept for high-performance textile-based TENGs and expand their application scope in human-machine interfacing.

## Abstract (web summary)

Low power output and poor sensing ability are bottlenecks for the practical application of fabric-based triboelectric nanogenerators (TENGs). The authors develop a shape adaptable and highly resilient 3D braided TENG, which is endowed with enhanced power output and improved pressure sensitivity.

## Introduction

With the arrival of the fourth industrial revolution and 5G information age, a wave of advanced industries and multidisciplinary fields including the Internet of Things (IoTs), cloud computing, big data, and artificial intelligence (AI) are springing up a multitude of personal-oriented functional wearable electronics^1–5^. A pressing issue has arisen behind this boom that abundant and low-power wearable electronics require theoretically inexhaustible, easily accessible, and ecologically sustainable power supply systems^6–10^. Although rechargeable capacitors/batteries can realize short-term power supply, they are unable to meet the future sustainable and eco-friendly energy demand due to their inherent defects of limited capacity, short service life, high charging frequency, and potential security risks^11–13^. Thankfully, energy harvesting technologies, such as solar cells^14–16^, thermoelectric generators^17,18^, biofuel cells^19,20^, enable us to acquire electricity directly from our surroundings. However, their reliable operations rely on specific external conditions, such as sunlight, temperature, auxiliary catalysts, etc. Therefore, a more active and less environmental dependent energy acquisition method is still imperative for the development of wearable electronics.

Triboelectricity refers to the phenomenon that a material is charged through contact or friction with another material. With the coupling effect of contact electrification and electrostatic induction, triboelectric nanogenerators (TENGs) can convert widely distributed but always neglected mechanical energy into electricity, whose theoretical origin can be traced back to Maxwell’s displacement current^21–23^. Due to its low cost, universal availability, environmental friendliness, and high conversion efficiency, TENGs have broad application prospects in both wearable power supplies and versatile self-powered sensing. However, most of the current TENGs present planar constructions or occupy large spaces, leading to the loss of clothing aesthetics and even restricting normal human movements. In order to realize the large-scale application of TENGs on the human body, it is necessary to develop a more effective and convenient combination form. In recent years, the integration of functional electronic components and traditional wearable textile technologies has promoted the emergency of electronic textiles (e-textiles), which can realize the collection, storage, processing, transmission, and display of information/data^24–26^. Through flexible material selection and textile structure design, TENGs can be developed into a new type of e-textiles, which can be worn on the human body without burden. On the one hand, TENGs are easily designed as fibers or integrated into fabrics, which endows traditional textiles with the abilities of mechanical energy harvesting and self-powered sensing. On the other hand, textiles are breathable, wearing comfortable, structurally flexible, mechanically robust, suitable for low-cost and massive production, which provide a broad application platform and diversified implementation carriers for the development of TENGs. With the help of the developed textile-based TENGs, a self-powered wearable system that can achieve continuous, stable, and reliable operation without external power supply and deliberate working conditions is established^27–31^. However, TENG-based e-textiles are subject to the developmental dilemmas of low power output and poor sensing abilities^7,32^. Although nanomaterials or nanostructures have been incorporated in an attempt to deal with the above issues, it is still difficult to achieve widespread application due to their complex preparation process, high production cost, and poor durability^33–36^. A more effective and easily scalable design scheme is a vital necessity for developing TENG-based e-textiles with high working performance and marketability.

Herein, based on the three-dimensional (3D) five-directional braiding structure, a 3D braided TENG (3DB-TENG) as a new TENG-based e-textile is developed for biomechanical energy harvesting and multifunctional pressure sensing. With the multiaxial winding yarn as the axial yarn and the PDMS-coated energy yarn as the braided yarn, the 3DB-TENG with the merits of high flexibility, shape adaptability, structural integrity, machine washability, and excellent mechanical stability is realized through a facile, readily implementable, and industrially scalable four-step braiding technology. The numerous spatial frame-column structures formed between the rhombic braided braced frame and the axial core column create a wealth of contact-separation space, endowing the 3DB-TENG with good air permeability, remarkable compression resilience, enhanced power output, improved pressure sensitivity, and even the abilities of vibrational energy harvesting and subtle weight detection. Under 3 Hz loading frequency and 20 N applied force, the 3DB-TENG can achieve an open-circuit voltage of 90 V and a peak power density of 26 W m^−3^, which can light up hundreds of LEDs, charge various commercial capacitors, and power miniature low-power electronics. In addition, a wireless intelligent footwear system for human motion monitoring, and a self-powered identity recognition carpet for safeguarding entrance are designed and demonstrated. Therefore, the developed 3DB-TENG as an e-textile with high-power output and high-pressure sensitivity shows broad application prospects in wearable power sources, wireless motion monitoring, and multifunctional human-machine interfacing.

## Results

### Fabrication of energy yarn

**Fig. 1:**
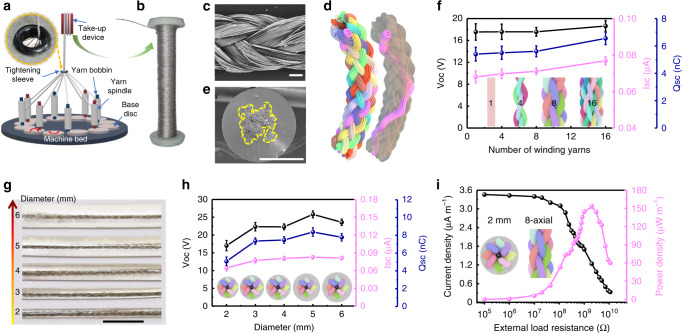
Structural design and performance analysis of the PDMS-coated energy yarn. **a** Schematic illustration of the multiaxial yarn winding machine. The enlarged photograph in its top left is the tightening sleeve. **b** Photograph of a bobbin wrapped with a continuous multiaxial winding yarn. **c** Surface morphology SEM image of a ten-axial winding yarn (scale bar: 200 μm). **d** Structural diagram of the ten-axial winding yarn. The purpose of different color design is to facilitate the observation of distribution and moving trend of a single yarn in the multiaxial winding yarn. **e** Cross-sectional SEM image of the PDMS-coated energy yarn (scale bar: 1 mm). **f** Effect of the number of winding yarns on electrical output performance of the PDMS-coated energy yarn. **g** Photograph of the PDMS-coated energy yarns with different diameters (scale bar: 10 mm). **h** Diameter-dependent electrical output performance of the PDMS-coated energy yarn. **i** Dependence of current density and power density of the PDMS-coated energy yarn with 2 mm diameter and 8 axial winding yarn on the external resistances under the tapping frequency of 3 Hz. The error bar indicates the range within a standard deviation. Source data are provided as a Source Data file.

It is well known that performance of TENGs depend largely on the selection of conductive and dielectric materials. In this study, commercial silver-plated nylon yarn is chosen as the electrode due to its low cost, easy access, and maturity in industrial production. Flexible yet tough PDMS elastomer is adopted as the dielectric material, owing to its inherent biocompatibility, excellent water resistance, high mechanical durability, and strong tendency to gain electrons. In order to improve the electrical conductivity and mechanical robustness of yarn electrode, a multiaxial winding method is adopted. As illustrated in Fig. 1a, several conductive yarns are drawn from the bobbins on the corresponding spindles and then interwoven with each other at the tightening sleeve. The reciprocating motion of spindles from one disc to another on the chassis contributes to the interweaving of yarns. With the aid of the top take-up device, the multiaxial winding yarn can be continuously and uniformly wound on the roller without length limitations (Fig. 1b). To illustrate the explanation, the photograph of the multiaxial yarn winding machine is displayed in Supplementary Fig. 1. It is noteworthy that the multiaxial winding method is revolutionary in the e-textile field which provides a facile, fast, efficient, and scalable approach to fabricating functional yarns with excellent electrical and mechanical properties. The surface morphology of a ten-axial winding yarn is observed by scanning electron microscopy (SEM, Fig. 1c), indicating that a single yarn moves forward in a spiral path along the axial direction and interweaves with others throughout the entire cross section (Fig. 1d). The electrical conductivity and mechanical properties of the multiaxial winding yarn are analyzed under different number of winding yarns. It is found that the cross-sectional area of the multiaxial winding yarn increases with the increasing of the number of winding yarns (Supplementary Fig. 2), resulting in the decrease of electric resistance (Supplementary Fig. 3), whereas the increase of the tensile modulus (Supplementary Fig. 4). With the multiaxial winding yarn as the electrode, energy harvesting yarn (or energy yarn) is obtained by coating PDMS evenly and conformally on its surface^27,30^. As shown in Fig. 1e, the multiaxial winding yarn highlighted with a yellow dashed line is well wrapped in the central zone of the outer circular PDMS elastomer. The effect of structural parameters, such as the number of winding yarns and the diameter of PDMS-coated energy yarn, on the electrical output are investigated. The test conditions for the electrical output of the PDMS-coated energy yarn are exhibited in Supplementary Fig. 5. Due to that the conductivity of the inner yarn electrode increases with the number of winding yarns, the electrical output capability of PDMS-coated energy yarn is also slightly enhanced (Fig. 1f and Supplementary Figs. 6, 7). It is worth noting that the coordination and balance between increasing electrical output and reducing the number of winding yarns can be well achieved in textile manufacturing (Supplementary Note 1). The diameter-dependent electrical output performance of PDMS-coated energy yarns is also compared (Fig. 1g). The result shows that the electrical output presents a trend of first rising and then declining with the increase of diameter, which can be attributed to the variation of the contact area between PDMS and the external substrate (Fig. 1h and Supplementary Fig. 8). The power density of PDMS-coated energy yarn is further calculated by connecting it in series with various external resistances. For example, when the applied frequency is 3 Hz, the instantaneous peak power density of the PDMS-coated energy yarn with 2 mm diameter and 8-axial yarn electrode can achieve the highest value of 150 μW m^−1^ at an external load of 2 GΩ (Fig. 1i). After a comprehensive trade-off of power output and total thickness, the PDMS-coated energy yarn with 8-axial yarn electrode and 2 mm diameter was hereafter chosen as the braided yarn to fabricate the 3DB-TENG (Supplementary Fig. 9).

### Structural characteristics

**Fig. 2:**
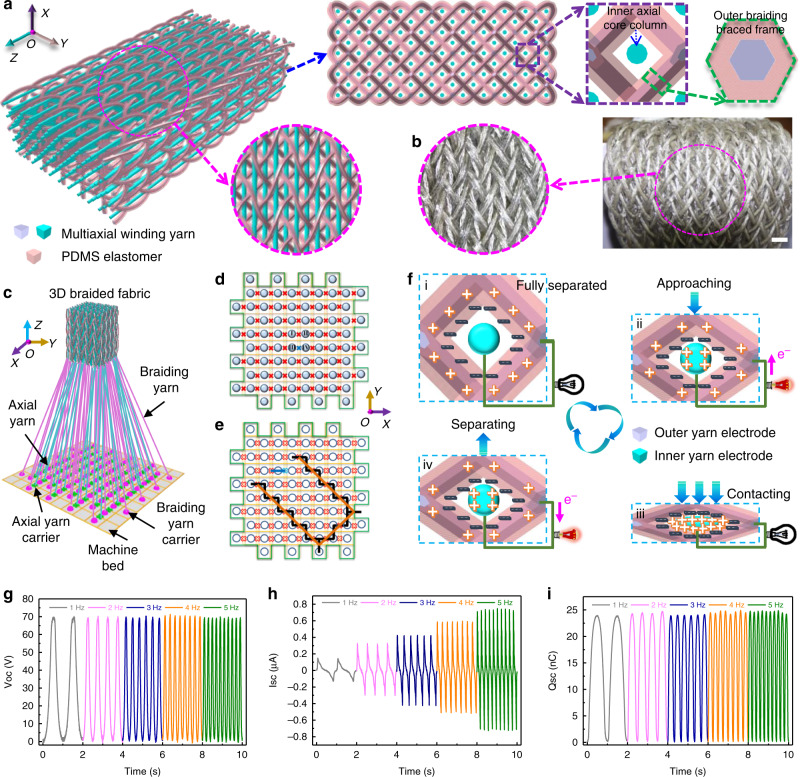
Structural characteristic, operational principle, and output performance of the 3DB-TENG. **a** Structural characteristic of the 3DB-TENG, which includes the outer braided braced frame and the inner axial core column. **b** Photograph image of the 3DB-TENG (scale bar: 1 cm). **c** Schematic illustration of the 3D four-step rectangular braiding technology. **d** Distribution of yarn carriers on the machine bed. The braided yarn carriers and the axial yarn carriers are marked with “*O*” and “*X*” symbols, respectively. **e** Traveled trajectories of one yarn carrier and its suspended yarn. The movement routes of one braided yarn and its yarn carrier are highlighted with an orange line and a black line, respectively. Moreover, the walking paths of one axial yarn and its yarn carrier are indicated with a blue line. **f** Schematic illustration of the working principle of the 3DB-TENG in a vertical contact and separation mode. **g–i** Electrical output performance of the 3DB-TENG under different loading frequencies (1–5 Hz), including (**g**) V_OC_, (**h**) I_SC_, and (**i**) Q_SC_. Source data are provided as a Source data file.

With the PDMS-coated energy yarn as the braided yarn and the eight-axial winding yarn as the axial yarn, the 3DB-TENG is fabricated through a four-step rectangular braiding technology in a self-developed 3D braiding machine. The topological structure and actual photograph of the 3DB-TENG are exhibited in Fig. 2a, b respectively, from which the 3D spatial configuration, interwoven frame structure, and yarn running trajectory can be observed. The braided yarn runs through the cross section and travels forward along the axial direction, interweaving both with each other as well as with the axial yarns through position conversion^37^ (Supplementary Fig. 10). In addition, the orientation of the braided yarn is not disordered but follows four basic directions, constructing numerous spatial rhombus-shaped braced frames. The axial yarn as the inner core column is located in the center of the braced frame, which can be regarded as the fifth direction. Based on the five orientations, a frame-column structure is established between the outer braided braced frame and the inner core column, which provides enough contact and separation space for them. The 3D four-step braiding process is achieved by the periodic row and column track movements of the yarn carriers on the machine bed in the *X* and *Y* direction, respectively (Fig. 2c). The arrangement of the carrier on the machine bed reflects the cross section of the fabric. The braided yarn carriers are arranged along both the row and column directions, while the axial yarn carriers are only located between the braided yarn carriers in rows. Assuming that the number of rows and columns of the braided fabric is m and n respectively, the total number of braided yarns will be m × n + m + n, where m × n and m + n are the number of the internal and external braided yarns, respectively. Moreover, the total number of axial yarns can be calculated as m × n. One end of yarn is hooked on the corresponding carrier, the other of which is connected to the finished fabric (Fig. 2c). The movement of the carrier promotes the interweaving of yarns, which will be further transferred to the top through a certain jamming action. The schematic diagram of the machine bed in the *XOY* plane is presented in Fig. 2d, in which the symbols of “*O*” and “*X*” refer to the braided yarn carriers and the axial yarn carriers, respectively. There are four movement steps in one machine cycle and the yarn carrier moves to at most one position with each step. The motions of yarn carriers in a complete four-step braiding cycle are described in Supplementary Fig. 11 and Supplementary Note 2. It is noteworthy that although the overall layout of yarn carriers on the machine bed is consistent with the original state, their actual locations have altered. As shown in Fig. 2e, the tracks traveled by one braided yarn and its connected yarn carrier after multiple braiding cycles are drawn with orange and black lines, respectively. It is found that the braided yarn carrier moves along a zig-zag path in the alternating movement of rows and columns. According to the principle of least squares, the trace of braided yarn in the cross section will be the connecting line from the mid-point of the yarn carriers^38^. The axial yarn carrier moves only in the *X* direction and returns to the original pattern after one four-step braiding cycle, as marked with a blue line in Fig. 2e. Therefore, it always moves in the connecting line between the carriers throughout the machine cycle, as does the axial yarn^39^. As these steps continue, the braided yarns move throughout the cross section and interlace with the axial yarns to form a fabric with structural integrity.

### Working mechanism and power output

The working principle of the 3DB-TENG is illustrated in Fig. 2f, which is operated in a vertical contact and separation mode. One cycle of compression and release motion is equivalent to a contact-separation process between the outer braided braced frame and the inner axial core column. Considering that PDMS has stronger ability to gain electrons than silver, the surface of PDMS is prone to be negatively charged, while the outer silver-coated yarn electrode tends to be positively charged. At the beginning, based on the high resilience of the 3DB-TENG, the inner core column and the outer braced frame will be in the maximum separated state (i). Due to the lack of potential difference, there is no charge or current in this state. When a compressed load is applied to the 3DB-TENG, the positive charges will be gradually induced from the outer yarn electrode to the inner yarn electrode under the electrostatic induction (ii). The accumulated potential difference promotes the reverse flow of electrons, which in turn generates an instantaneous positive current. Once the 3DB-TENG is fully compressed, the negative triboelectric charges on the surface of PDMS are completely balanced by the positive electrostatic charges induced on the inner yarn electrode (iii). In this case, the electrical signals will be absent because the charges are offset by each other. However, due to the inherent features of the insulator, the accumulated charges will be kept for a period of time rather than entirely annihilated. If the pressure is removed, the 3DB-TENG will bounce up rapidly due to its high resilient braiding structure. The positive charges accumulated on the inner yarn electrode will flow back to the outer yarn electrode to compensate for the potential difference (iv). After the 3DB-TENG returns to the maximum relaxation state (i), the negative charges on the surface of PDMS are completely neutralized by the positive charges induced on the outer yarn electrode. Therefore, it can be found that a contact and separation process will generate an instantaneous alternating current through external loads. A theoretical model based on finite element simulation of COMSOL Multiphysics is established to quantitatively analyze the potential distribution of the 3DB-TENG in a contact-separation process (Supplementary Fig. 12). The frequency-dependent electrical output performance of the 3DB-TENG, including open-circuit voltage (V_OC_), short-circuit current (I_SC_), and short-circuit charge transfer (Q_SC_) is measured under the compression load of 20N (Fig. 2g–i). It is found that a relatively stable V_OC_ and Q_SC_ (70 V and 25 nC, respectively) and a gradually increasing I_SC_ (from 0.16 to 0.7 μA) are observed as the frequency increases from 1 to 5 Hz. The inconsistent variation of electrical output characteristics with the loading frequency is explained in Supplementary Fig. 13 and Supplementary Note 3.

**Fig. 3:**
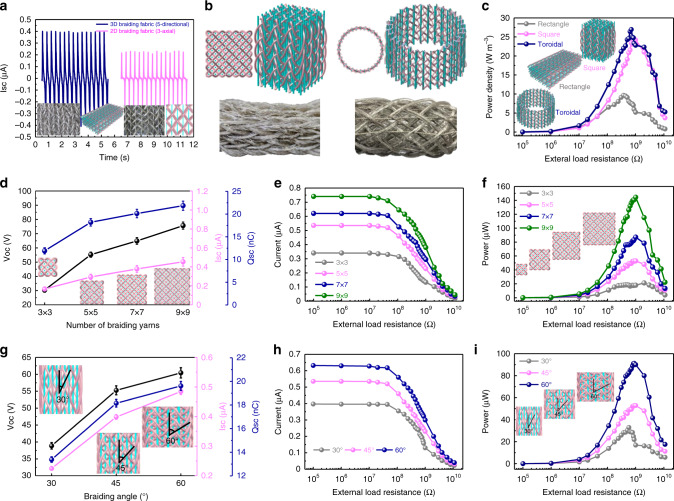
Effect of braiding parameters on the electrical output performance of the 3DB-TENG. **a** Comparison of the I_SC_ between the 3DB-TENG and a multilayered 2D triaxial braided TENG fabric. Their respective photographs and structural diagrams are displayed at the bottom. **b** Structural diagrams (top) and photograph images (bottom) of the 3DB-TENGs with square-shaped (left) and toroidal-shaped (right) cross sections. **c** Comparison of the power densities of the rectangle-shaped, square-shaped, and toroidal-shaped 3DB-TENGs. **d** Effect of the number of braided yarns (3 × 3, 5 × 5, 7 × 7 and 9 × 9) on the power output of the 3DB-TENGs. **e**, **f** Comparisons of the (**e**) current and (**f**) power of the square-shaped 3DB-TENGs with different number of braided yarns. **g** Effect of the surface braiding angles (30°, 45°, and 60°) on the electrical output of the 3DB-TENGs. **h**, **i** Comparisons of the (**h**) current and (**i**) power of the square-shaped 3DB-TENGs with different surface braiding angles. The error bar indicates the range within a standard deviation. Source data are provided as a Source data file.

The enhanced electrical output of the 3DB-TENG is verified by comparing with a multilayered 2D TENG fabric, which is designed by stacking several 2D triaxial braided fabrics along their thickness direction to keep its dimensions the same as that of the 3DB-TENG (Supplementary Fig. 14 and Supplementary Note 4). The result shows that the I_SC_ of the 3DB-TENG is nearly twice than that of the multilayered 2D TENG fabric, indicating that the special 3D braided structure with more contact-separation spaces can effectively improve the total power output (Fig. 3a). Shape adaptability is another remarkable feature of the 3DB-TENG, which can be adjusted according to the arrangement of yarns on the machine bed. In addition to the rectangular shape, the cross section of the 3DB-TENG can also be designed as square shape and toroidal shape (Fig. 3b). By comparing their electrical outputs (Supplementary Fig. 15), it can be found that the optimal external resistance of the three types of 3DB-TENGs are basically the same, which can be attributed to the same test conditions (Supplementary Note 5). In addition, the output power density of the toroidal shape is the highest while that of rectangular shape is the lowest (Fig. 3c). The maximum peak power density can be used to evaluate the figure of merit of the 3DB-TENG (Supplementary Note 6), which can reach 26 W m^−3^ under the loading frequency of 3 Hz and the applied force of 20 N. The toroidal-shaped 3DB-TENG with an elastic hollow configuration has better resilience than the others, which can provide greater separation distance under the same loading conditions. Moreover, the higher power output of the square shape than that of rectangular shape may be due to more yarns being distributed in the crosswise direction. To verify this assumption, the effect of the number of braided yarns on the power output is investigated. It is found that the electrical output of 3DB-TENG is enhanced with an increased number of braided yarns (Fig. 3d–f and Supplementary Fig. 16), verifying that the more yarns there are in the crosswise direction, the higher electrical output will be. The braiding tightness is reflected by the surface braiding angle (β) between the axis of surface braided yarn and the *Z*-axis^40^ (Supplementary Fig. 17). As depicted in Fig. 3g–i and Supplementary Fig. 18, the larger the β is, the better electrical output of the 3DB-TENG will be, which can be explained that more interwoven yarns are involved in the unit length of larger β.

### Pressure sensitivity, stability, and washability

**Fig. 4:**
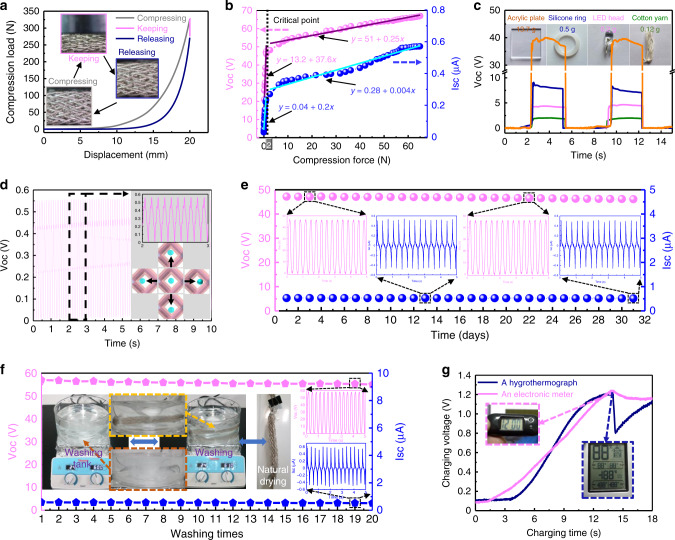
Pressure sensitivity, long-term stability, and washability of the 3DB-TENG. **a** Load-displacement curve of the 3DB-TENG in the process of compression, retention, and release. **b** Variations of V_OC_ and I_SC_ of the 3DB-TENG as a function of applied forces. **c** Pressure sensitivity of the 3DB-TENG to different objects. **d** Vibrational energy harvesting ability of the 3DB-TENG under a vibrational frequency of 10 Hz. The images inserted at the top and bottom are the enlarged V_OC_ output and the vibrational energy harvesting principle, respectively. **e** Analyses of the stability and durability of the 3DB-TENG by continuous test for nearly one month. **f** Analysis of the washability of the 3DB-TENG after 20 times washing. The inserted photos are the cultivated domestic washing environment. **g** Charging voltage curves of the 3DB-TENG in the process of powering electronic devices. The inserts are the photos of electronic devices under the operational state. Source data are provided as a Source data file.

The load-displacement curve of the 3DB-TENG during compression, retention and release is shown in Fig. 4a. The corresponding loading process is demonstrated in Supplementary Fig. 19 and Supplementary Movie 1. It can be observed that the difference between the compression ascent curve and the release rebound curve is small even if the fully compressed state lasts for 10 s. In addition, the compression resilience coefficient during this loading process can reach ~ 60 %, which further verifies its remarkable compression resilience (Supplementary Fig. 20 and Supplementary Note 7). The electrical output of the 3DB-TENG as a function of loading force is studied to explore its pressure sensitivity. As shown in Fig. 4b, both V_OC_ and I_SC_ present approximate bilinear growth trends with the increase of loading forces. In addition, with 2 N as the critical point, the slope of the first line is much larger than that of the second one. In the first stage, the increase of pressure will cause a significant change of gap distance, leading to larger electrical output variations. However, when the pressure continues to increase to the second stage, the gap distance has reached the maximum, and further increasing of pressure will only work for enhancing the effective contact area (Supplementary Note 8). The bilinear increasing behaviors with different slopes reflect the excellent pressure sensitivity of the 3DB-TENG, which can respond to slight weight changes. For example, the 3DB-TENG can repeatedly and steadily distinguish electrical signal differences even if the mass variation is less than 0.1 g (Fig. 4c). With high compression resilience and high pressure response sensitivity, the 3DB-TENG also has the ability to harvest vibrational energy. The adopted vibration test platform (Supplementary Fig. 21) and detailed test conditions for vibrational energy harvesting are introduced in Supplementary Note 9. It can be found that since both ends of the 3DB-TENG are fixed on the bracket of the electrodynamic shaker, its actual activation area is the part above the support frame (Supplementary Fig. 22). Under 10 Hz vibration frequency and 5 mm vibration amplitude, the 3DB-TENG can achieve an V_OC_ of 550 mV (Fig. 4d). The potential mechanism of vibrational energy harvesting can be attributed to the contact and separate movement between the inner core column and the outer braced frame under the synergistic effect of the shaker and the inertia (lower right in Fig. 4d). In addition, the long-term stability of the 3DB-TENG is discussed, and the corresponding test conditions and methods are introduced in Supplementary Note 10. The result shows that the V_OC_ and I_SC_ have no significant reduction during one month of cyclic loading (Fig. 4e and Supplementary Fig. 23), which exhibits good mechanical robustness and working durability. Moreover, machine washability is also a basic requirement for textiles in their actual application. In this study, we have cultivated a simulated domestic laundering environment with household detergents and magnetic stir bars added. The detailed introduction of the washing process and conditions are summarized in Supplementary Note 11. It turns out that the electrical output performance of the 3DB-TENG can be well maintained without significant degradation even after 20 times washing (Fig. 4f). The satisfactory functional and washing durability of the 3DB-TENG are due to its stable material properties and integrated braided structure.

Several applications of the 3DB-TENG are demonstrated in terms of wearable power supply and self-powered sensing. First of all, as a wearable power source, the 3DB-TENG can directly light up hundreds of LEDs simply by tapping it by hand (Supplementary Fig. 24 and Supplementary Movie 2). In addition, the electrical output can be rectified and stored in capacitors or batteries for later use. The charging abilities of the 3DB-TENG under different loading frequencies (1–5 Hz) and capacitance capacities (1–47 μF) are compared (Supplementary Fig. 25), which shows that the increase of frequency or the reduction of capacitance is beneficial to accelerating the charging speed. The stored electricity can sustainably power miniature wearable electronics, such as hygrothermographs and electronic meters (Supplementary Movie 3). The corresponding charging voltage curves are recorded in Fig. 4g, indicating that the device will start to work once the threshold voltage is reached, and can operate sustainably under continuous charging conditions. Therefore, the developed 3DB-TENG is likely to be used as a wearable power source in the future.

### An intelligent footwear system

**Fig. 5:**
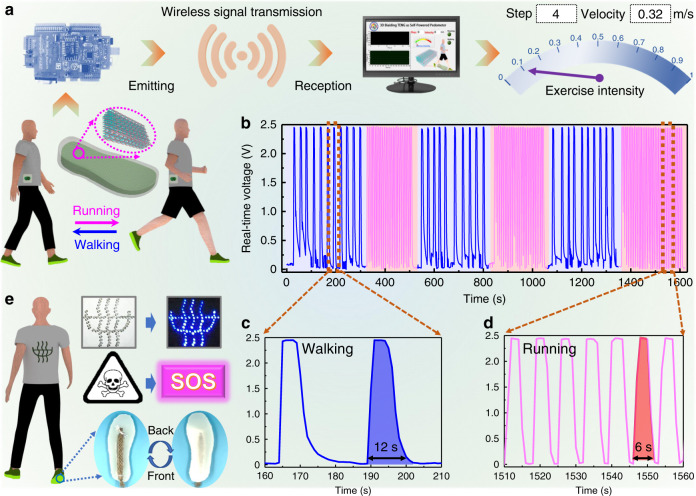
An intelligent footwear system for human motion monitoring and remote emergency rescue. **a** Flowchart of the intelligent footwear system for real-time human motion monitoring, including a signal acquisition system, a wireless transmission module, a data processing program, and a software output interface. **b** Real-time voltage signals of the intelligent footwear system during the alternating process of walking (blue) and running (magenta). **c**, **d** Enlarged voltage signals of walking (**c**) and running (**d**) states, in which the interval period of each step is 12 s and 6 s, respectively. **e** Demonstrations of the intelligent footwear system in night safety protection and remote emergency rescue. Source data are provided as a Source data file.

Compared with the power supply, self-powered sensing allows the 3DB-TENG to be used in a wider range of fields. Firstly, an intelligent footwear system for human motion monitoring and remote emergency rescue is developed. As shown in Fig. 5a, the system for real-time human motion monitoring includes a signal acquisition system, a wireless transmission module, a data processing program, and a software output interface. In addition, the actual integrated photograph of the system is exhibited in Supplementary Fig. 26. It is found that the 3DB-TENG is designed as a rubber encapsulated sole, which can not only isolate environmental interferences as well as protect against contamination, but also convert human motions into real-time voltage signals. The voltage signals can be captured and transmitted through the wireless transmission module based on a single chip microcomputer, which is attached to human body for convenience. The self-developed data processing program based on LabVIEW will simultaneously calculate, analyze, and judge the received wireless signals. The step number, average velocity, and exercise intensity will be presented and reported on the software output interface (Supplementary Fig. 27). The intelligent footwear system in human motion monitoring is demonstrated in Supplementary Movie 4, from which the corresponding voltage output signals are recorded in Fig. 5b. It is found that voltage signal with an approximate trapezoidal characteristic has good repeatability and stability. When the sole is stepped down, an instantaneous forward voltage signal is generated, which will reduce rapidly and finally approach zero once the foot is raised. Therefore, a motion step corresponds to a forward voltage signal, and the total motion step number (n) can be calculated according to the number of voltage peaks. The travelled distance (s) and motion velocity (ν) can be obtained based on the equations s = n × L and ν = L × ƒ, where L and ƒ represent the length and frequency of steps, respectively. In addition, the motion frequency is an important index to distinguish walking from running. The voltage signals can be divided into two parts, i.e., the sparse part highlighted in blue and the dense part marked in magenta, which corresponds to the walking and running states, respectively (Fig. 5b). There is a significant difference in the frequency of walking and running, which hold for 12 and 6 s each step, respectively (Fig. 5c, d). The exercise intensity depends on the motion velocity, which varies between 0 (static) and 1 (sprinting). In addition to monitoring human movement, the system can also be used for remote emergency rescue to minimize or avoid potential hazards. As shown in Fig. 5e, it can light up safety indication signs on the human body in dark environments to distinguish individuals to passing drivers. Moreover, it can send distress signals remotely and wirelessly when potential hazards approach.

### A self-powered identity recognition carpet

**Fig. 6:**
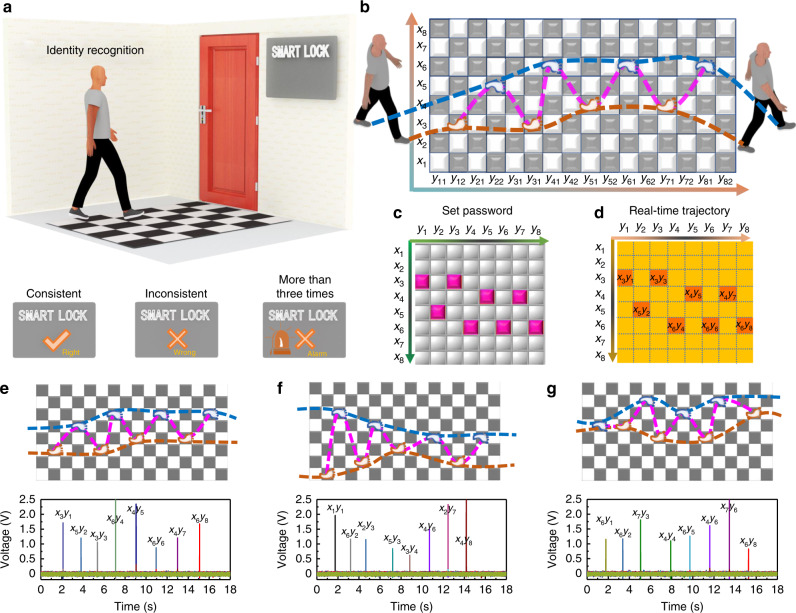
A self-powered identity recognition carpet for safeguarding entrance and early warning of intrusion. **a** Schematic illustration of the self-powered identity recognition system. There will be three states displayed on the screen, i.e., “√”, “×”, and “×” accompanied by an alarm indicator. **b** Schematic illustration of the human walking trajectories on the identity recognition carpet. The magenta, blue, and brown dotted lines represent the walking routes of the whole person, the left foot, and the right foot, respectively. Moreover, the small blue and brown foot symbols refer to the left foot and the right foot, respectively. **c**, **d** Schematic illustrations of (**c**) the set password path and (**d**) the real-time walking path. **e**–**g** Real-time walking trajectories on the identity recognition carpet, which correspond to the path with (**e**) the correct password, (**f**) the incorrect password for the first time, and (**g**) the incorrect password for more than three consecutive times when evaluated against the set password path. The voltage signals of 64 sensing blocks are presented below the corresponding walking trajectories and the positions of the stepped sensing blocks are marked above each curve. Source data are provided as a Source data file.

In the modern information society, access security has become an unavoidable issue, especially in the military and commercial fields. It is an important guarantee to maintain internal security and prevent foreign invasion by developing a simple, efficient, and self-powered visitor identification system. To this end, a self-powered identity recognition carpet system is designed for safeguarding entrance and early warning of intrusion (Fig. 6a), which includes a self-powered energy harvesting carpet, a synchronous multichannel acquisition module, and a real-time data analysis and interface output platform (Supplementary Figs. 28 and 29). The self-powered identification carpet consisting of 128 equally-sized black and white checkered square blocks is placed in front of the smart door. The area of each block is designed as 20 × 20 cm^2^ based on the size of normal feet. The 3DB-TENG fabrics are sewn in horseshoe shape at the back center of the black blocks, which constitutes 64 mutually independent sensing regions (Supplementary Fig. 30). The extraction electrodes of the 3DB-TENG are connected with the corresponding acquisition channels by standard flexible insulated electronic wires. Therefore, each sensing region is connected to its own receiving channel so that they are independent of each other. The purpose of the white blocks is to reduce the interference between adjacent sensing regions and facilitate visitors to walk. The developed software output interface includes the set password path, the real-time walking path and output signal, and the switchable judgment states (Supplementary Fig. 31). The data analysis and processing method of the generated voltage signals are explained in Supplementary Fig. 32 and Supplementary Note 12. In short, we first eliminate the interference signals by setting the threshold voltage, and then determine the final activated position by comparing the maximum value of the remaining signals. In fact, there will be three states displayed on the interface, i.e., the correct password identity, the wrong password identity, and the wrong identity accompanied by an alarm ringing if the incorrect passwords are entered over three times in a row. When a visitor walks through the carpet, his walking trajectories will be recorded simultaneously. As shown in Fig. 6b, the walking paths of an entire person, their left foot and right foot are outlined with magenta, blue, and brown dotted lines, respectively. The blocks stepped on are highlighted with small foot symbols, which are divided into blue and brown according to left foot and right foot assignments, respectively. For the convenience of displaying positions, the row and column of the carpet are marked as *x*_i_ and *y*_ij_ (i = 1, 2,……, 8, j = 1 and 2), respectively. Real-time walking trajectories are mapped on the interface (Fig. 6c) and matched with the set password path (Fig. 6d). Please note that the white regions have been removed from the interface for ease of identification. If the path is consistent with the set one, the correct information will be displayed on the screen and the door will be opened (Fig. 6e). However, the door will remain closed when an inconsistent validation of authentication is reported (Fig. 6f). What’s more, if there are more than three consecutive inconsistencies, the danger intrusion alarm will be issued at the same time (Fig. 6g). The voltage signals of the 64 sensing regions are presented below the corresponding walking trajectories, which are further marked based on their positions on the carpet. In practice, there may be some unexpected situations, such as stepping on the junction of two black blocks (Supplementary Fig. 33), the hysteresis effect of signal acquisition and transmission, the influence of signal crosstalk, and potential confidentiality issue (Supplementary Fig. 34). In this study, we have also taken some measures to reduce or avoid them, which are discussed in Supplementary Note 13. The continuous voltage signal of a single sensing block is presented in Supplementary Fig. 35, showing good consistency and stability. Moreover, the repeatability and reliability in terms of walking path identification are also investigated. The result shows that voltage signals with the same trend are obtained on the set path even after 10 repeated tests, in which the accuracy is close to 100% (Supplementary Fig. 36). The application of the identity recognition carpet in safeguarding entrances and early warning of intruders is demonstrated in Supplementary Movie 5. The developed self-powered identity recognition carpet with merits of high recognition accuracy and good stability has great prospects for application in future identity authentication, access control system, and gait analysis.

## Discussion

In summary, we have developed a shape adaptable and highly resilient TENG-based 3D e-textile through a readily implementable and industrially scalable four-step braiding technology for wearable power supply and multifunctional pressure sensing. Due to the optimized material selection and well-designed 3D five-directional braiding structure, the 3DB-TENG has been qualified with high flexibility, good structural integrity, high compression resilience, enhanced power output, improved pressure sensitivity, excellent working durability, and machine washability. In addition, with the help of a large number of spatial frame-column structural networks constructed between the outer braided yarn and the inner axial yarn, the 3DB-TENG also has the abilities to harvest vibrational energy, power miniature wearable electronics, and respond to subtle weight variations. Moreover, two new forms of human-machine interactive applications, including an intelligent shoe for human motion monitoring and a self-powered identity recognition carpet for safeguarding entrance, are demonstrated to verify its superior pressure sensing performance. This work provides a new design concept of 3D five-directional braiding structure and several innovative application occasions for the TENG-based e-textiles with high power output and high pressure sensitivity, which may push forward progress in wearable energy harvesting, self-powered sensors, human-machine interfacing, and artificial intelligence.

## Methods

### Preparation of multiaxial winding yarn

Commercial silver-plated nylon yarn (nominal diameter: 180 μm, resistance < 100 Ω cm^−1^, LessEMF.com) was chosen as the electrode. The multiaxial winding yarn was prepared on a high-speed rope braiding machine (Fig. 1a). Firstly, commercial silver-coated nylon yarns were wound on the bobbins, which were fixed on the spindles. The spindle was anchored to a base disc that was fastened to the machine bed. The continuous supply of yarn was achieved by the rotation of the bobbin on the spindle. The yarn tension during the feeding process could be adjusted by the tension device. The yarn interweaving was realized due to the interaction between the transfer of spindle from one disc to another and the restraint of the tightening sleeve. The continuous winding of the multiaxial yarn was achieved by the top take-up device. The number of axes of the winding yarn depended on the number of feeding spindles.

### Preparation of energy yarn

Polydimethylsiloxane (PDMS) was selected as the flexible dielectric material. The energy harvesting yarn (or energy yarn) was prepared by conformally and uniformly coating PDMS on the surface of the above-prepared multiaxial winding yarn. Here, the method is also briefly introduced for better understanding. The multiaxial winding yarn was inserted into a circular plastic tube with the two ends of the yarn fixed in its center. The liquid PDMS solution was prepared by mixing the base monomer and the curing agent in a 10:1 weight ratio (Sylgard 184, Dow corning), then blending, and degassing in a vacuum for about 15 min to thoroughly remove bubbles. The obtained PDMS solution was injected into the tube until it was filled. After a period of standing, the PDMS got slightly viscous and then the tube was transferred to an oven. After curing at 80 °C for 2 h, the PDMS-coated energy yarn was obtained by removing the circular plastic tube.

### Fabrication of 3DB-TENG

The 3DB-TENG was fabricated with the PDMS-coated energy yarn as the braided yarn and the multiaxial winding yarn as the axial yarn in a self-developed 3D braiding machine based on a four-step rectangular braiding technology. In the four-step braiding process, the braided yarns interwoven regularly with each other through the row and column movements of the yarn carriers in the X and Y directions. The braided yarns and the axial yarns were connected with the corresponding yarn carriers that were fixed on the machine bed (Fig. 2c). Every machine braiding cycle consisted of four movement steps, in which each yarn carrier moved only one position per step. In the first step, the rows of the braided yarn carriers and axial yarn carriers moved one position horizontally in an alternating way. In the second step, only the braided yarn carriers moved one position alternately in the vertical direction, whereas all the axial yarn carriers stayed in their current locations without moving. The third and fourth steps reversed the movement of yarn carriers in the first and second steps, respectively. After the four moving steps, the yarn carriers would return to the initial distribution pattern, completing one machine cycle. Afterward, a certain jamming action was imposed on all the yarns to make them more or less closely intertwined. By repeating multiple braiding cycles, a 3DB-TENG with a controllable length was obtained. It is noteworthy that the cross-sectional shape and size of the 3DB-TENG could also be adjusted according to the distribution position and yarn count on the machine bed, respectively. In addition, the degree of interweaving tightness depended on the surface braiding angle.

### Characterization and measurement

The multiaxial winding yarn was prepared based on a high-speed rope braiding machine (Xuzhou Henghui Braiding Machinery Co., Ltd). Field emission scanning electron microscope (SEM, SU-8020, Hitachi) was used to characterize the surface morphology of the multiaxial winding yarn and the cross section of the PDMS-coated energy yarn. The mechanical tensile and compression tests were performed on a universal material tester (MTS, Model Insight 10). The diameter was determined by an electronic micrometer (733 Series Electronic Digital Micrometer, L.S. Starrett). The periodic contact and separation movements were applied by a commercial linear mechanical motor (LinMot E1100). A compression dynamometer (Vernier LabQuest Mini) was used to detect the applied force. The open-circuit voltage (V_OC_), short-circuit current (I_SC_), and short-circuit charge transfer (Q_SC_) were measured by an electrometer (Keithley, model 6514). The wired transmission of electrical signal was achieved by connecting yarn electrodes to the standard flexible insulated electronic wires fabricated with copper as the core and polyvinyl chloride (PVC) as the shell (model: RV 0.75 mm^2^), the two joint of which is fixed with conductive silver paste. The human motion data of the intelligent shoe were collected by a wireless motion monitoring system based on a My-ARM-DAQ board. A synchronous multichannel data acquisition card (PXIe-4300, National Instruments) with integrated signal conditioning was used for real-time data acquisition of the identification carpet.

## Supplementary information

Supplementary Information

Description of Additional Supplementary Files

Supplementary Movie 1

Supplementary Movie 2

Supplementary Movie 3

Supplementary Movie 4

Supplementary Movie 5

## Data Availability

All data needed to evaluate the conclusions in the paper are present in the paper and/or the Supplementary Information. Additional data related to this paper may be requested from the authors upon reasonable request. The source data underlying all figures can be found in the Source data file.
