# Supplementary material for: Shape adaptable and highly resilient 3D braided triboelectric nanogenerators as e-textiles for power and sensing
Source: Nat Commun. 2020 Jun 8;11:2868. doi: 10.1038/s41467-020-16642-6 (PMC7280288; doi:10.1038/s41467-020-16642-6)
Supplement: Supplementary file 1 — Supplementary Information [file 41467_2020_16642_MOESM1_ESM.pdf]

Supplementary Information

Shape adaptable and highly resilient 3D braided triboelectric nanogenerators as e-textiles for power and sensing

Dong *et al*.

Supplementary Figures

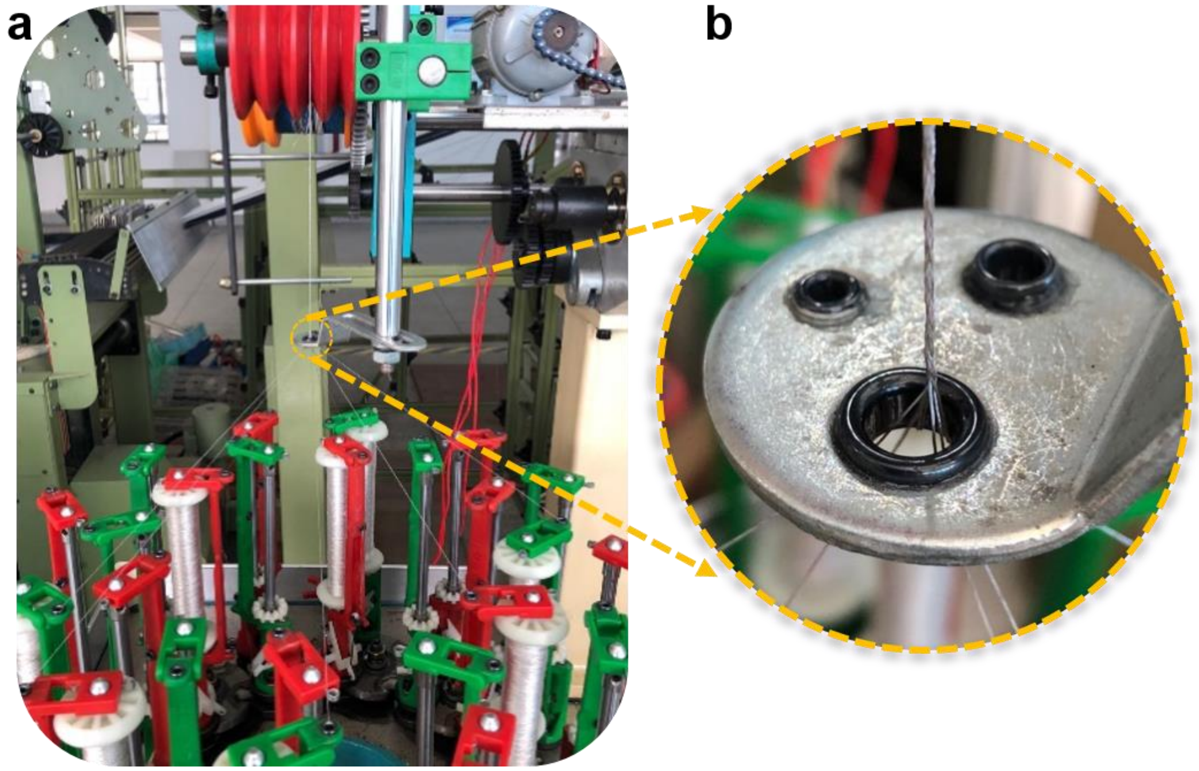

Supplementary Figure 1. Demonstration of the multiaxial yarn winding machine. a Photograph of the multiaxial winding machine. **b** Photograph of the enlarged tightening sleeve.

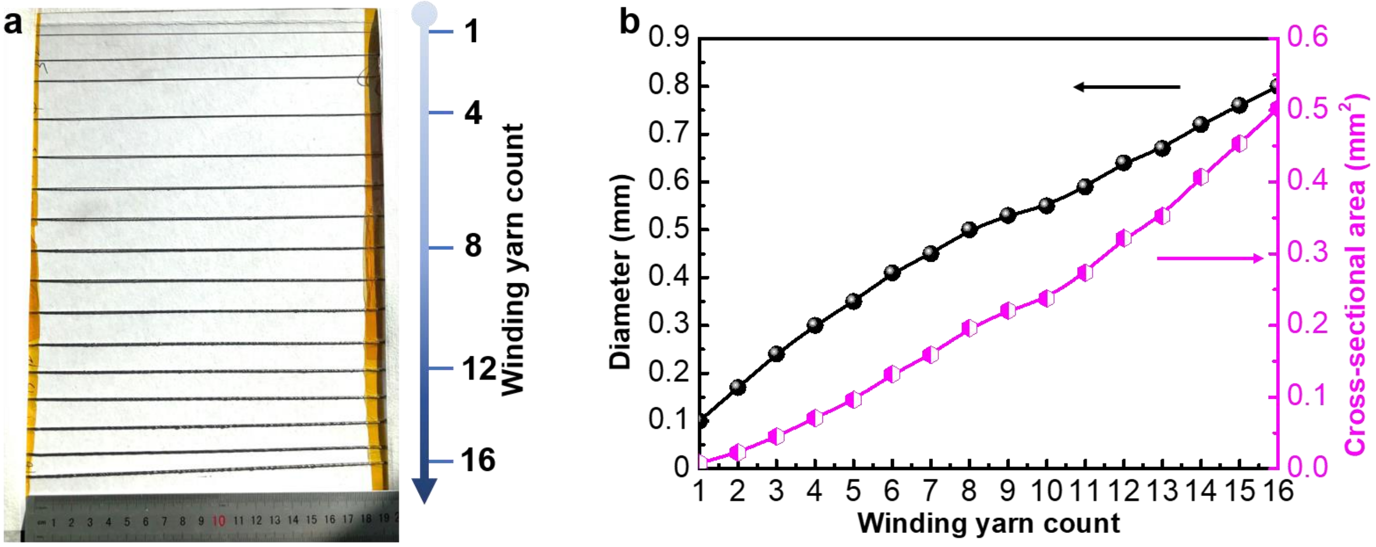

Supplementary Figure 2. Effect of the number of yarns on the diameter and cross-sectional area of the multiaxial winding yarns. a Photograph of multiaxial winding yarns with different yarn counts (1-16 from the top to the bottom). **b** The diameter and cross-sectional area of the multiaxial winding yarns under different yarn counts. We assumed that the cross section of the multiaxial winding yarn is approximately circular.

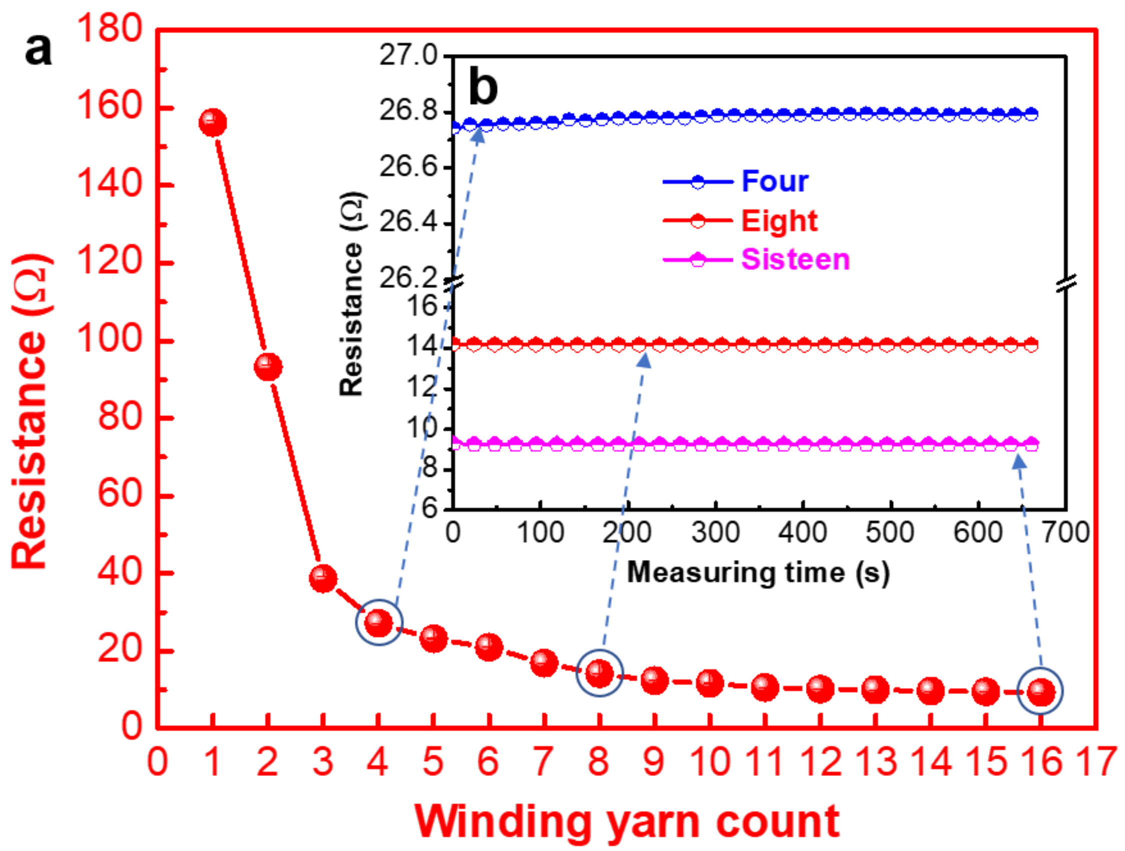

Supplementary Figure 3. Electrical conductivity of multiaxial winding yarn under different number of winding yarns. a Effect of the winding yarn count on the electrical conductivity of multiaxial winding yarns. **b** Analysis of the stability of electric resistance over a period of time (the measured distance is about 20 cm).

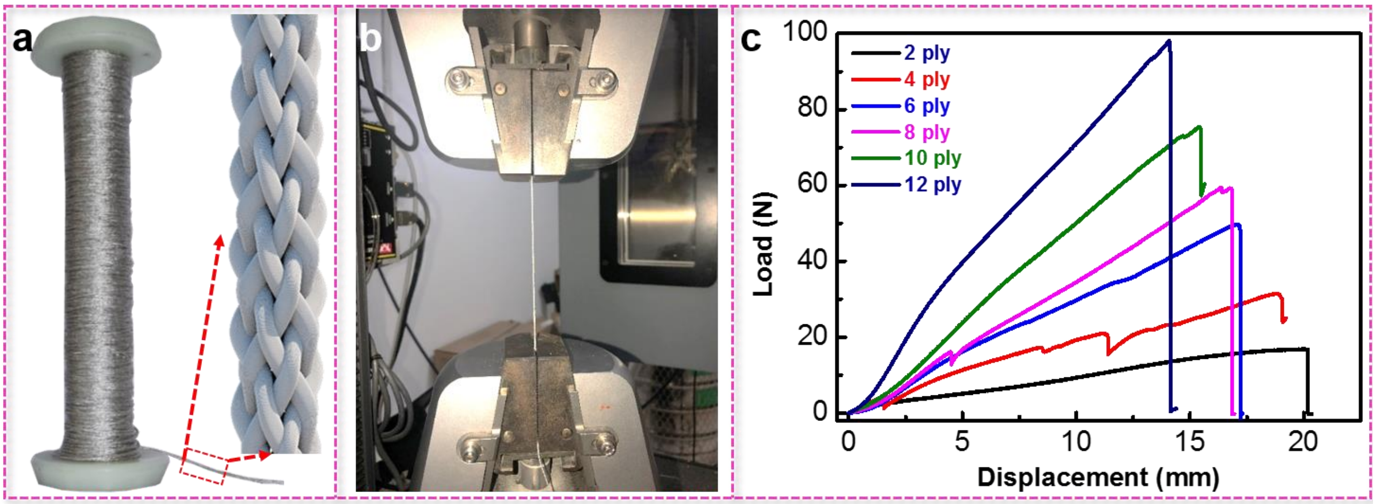

Supplementary Figure 4. Uniaxial tensile test of the multiaxial winding yarns. a Photograph of a bobbin wrapped with multiaxial winding yarn. The structural diagram of multiaxial winding yarn is enlarged on the right. **b** Photograph of the uniaxial tensile tester. **c** Load-displacement curves of the multiaxial winding yarns.

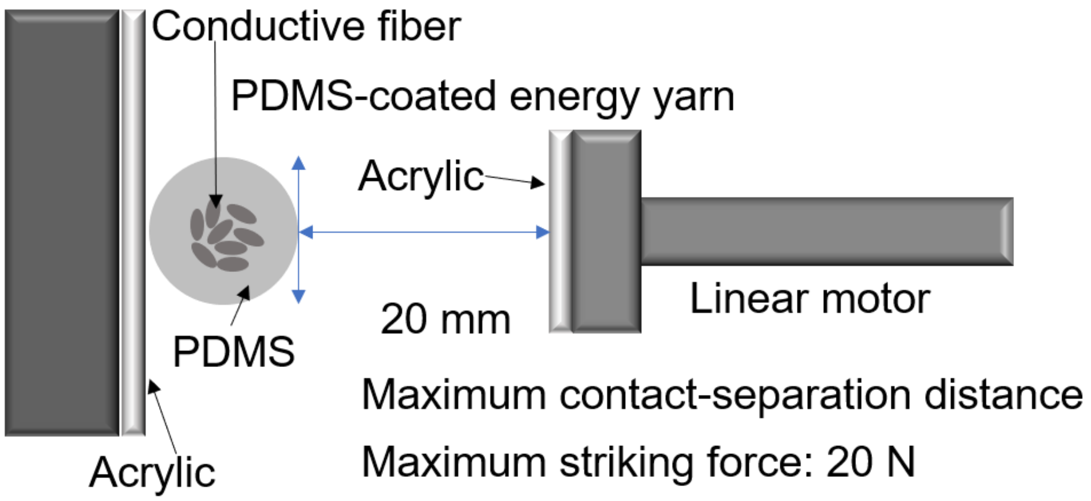

Supplementary Figure 5. Experimental apparatus and test conditions for the electrical output performance of the PDMS-coated energy yarns. The periodic contact-separation movements between PDMS-coated energy yarns and acrylic plates (external contact material) are imposed by linear mechanical motor (LinMot E1100). In addition, the striking force is 20 N, the maximum separation distance is approximately 20 mm, and the applied frequency is fixed at 1 Hz. In order to compare the effect of yarn numbers on the electrical output performance of the PDMS-coated energy yarn, the diameters of all the PDMS-coated energy yarns are kept at about 2 mm.

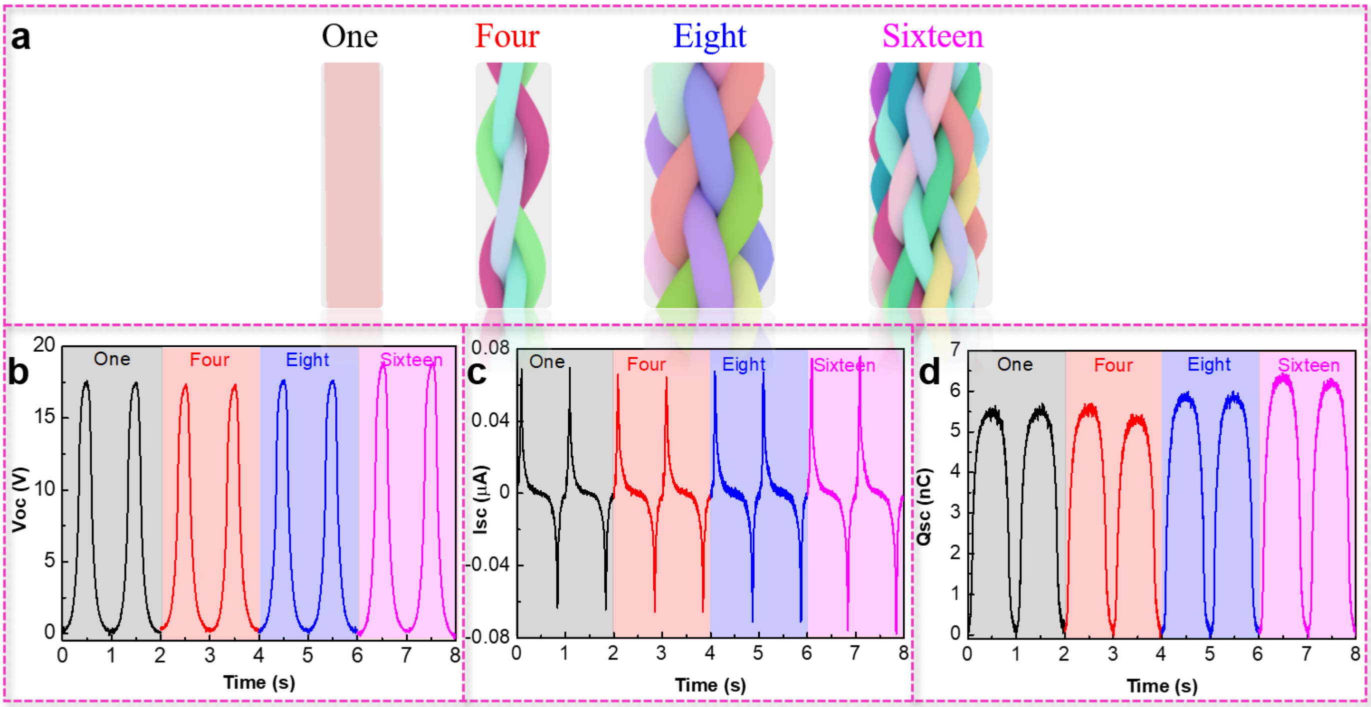

Supplementary Figure 6. Effect of the number of conductive yarns on the electrical output performance of the PDMS-coated energy yarns. a Schematic illustration of the conductive yarns with uniaxial, 4-axial, 8-axial, and 16-axial configurations. **b-d** Comparison of the electrical output performance of their corresponding energy yarns, including (**b**) open-circuit voltage (V_{OC}), (**c**) short-circuit current (I_{SC}), and (**d**) short-circuit charge transfer (Q_{SC}).

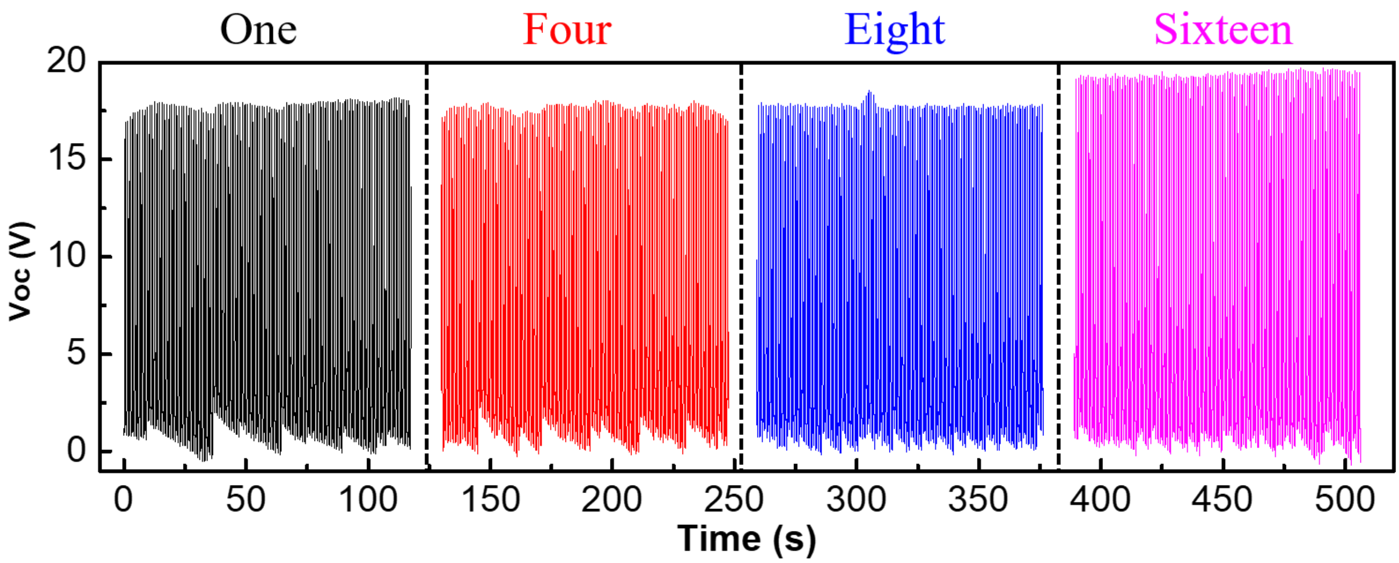

Supplementary Figure 7. Comparison of open-circuit voltage (V_{OC}) of the PDMS-coated energy yarns over a long period of time. The PDMS-coated energy yarns are prepared with different number of winding yarns (1, 4, 8 and 16).

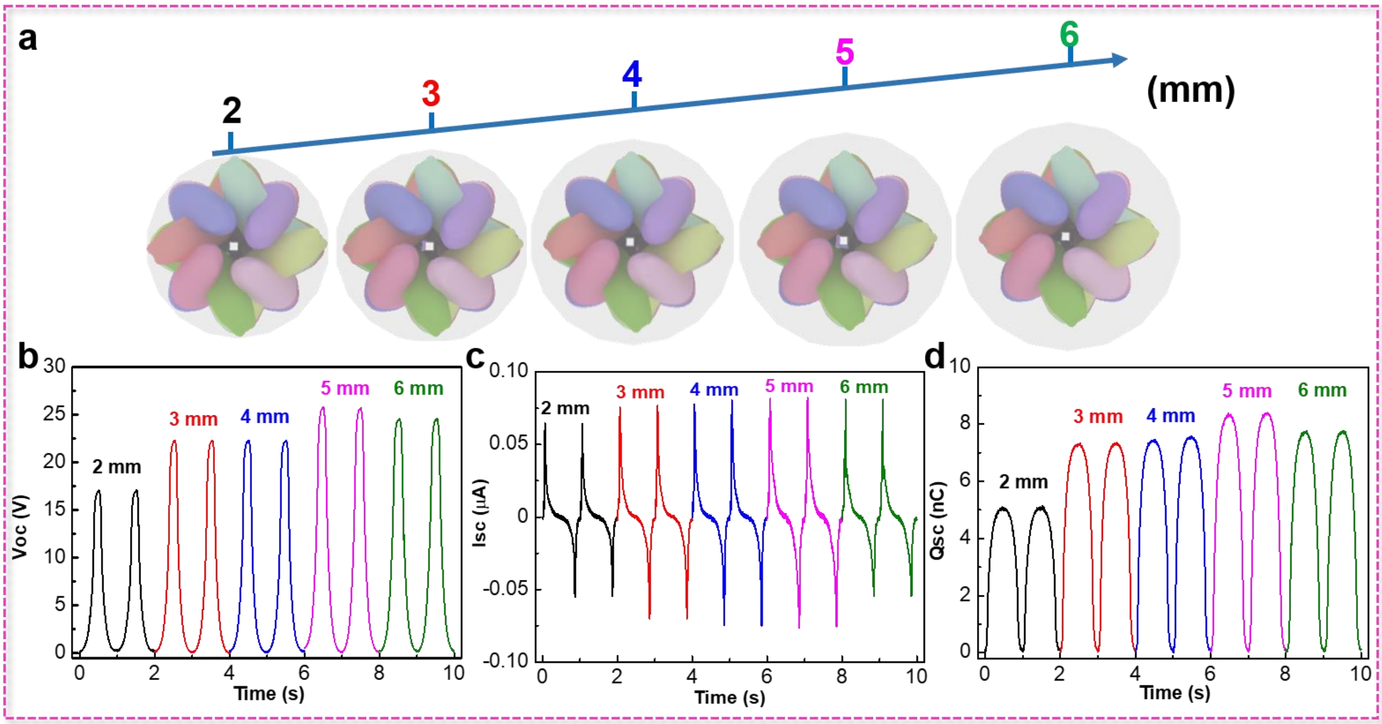

Supplementary Figure 8. **Effect of the diameter (or the coating amount of PDMS) on the power output performance of the PDMS-coated energy yarn. a** Schematic illustration of the PDMS-coated energy yarns with different diameters (2, 3, 4, 5 and 6 mm). **b-d** Diameter-dependent electrical output performance of the PDMS-coated energy yarns, including (**b**) V_{OC}, (**c**) I_{SC}, and (**d**) Q_{SC}.

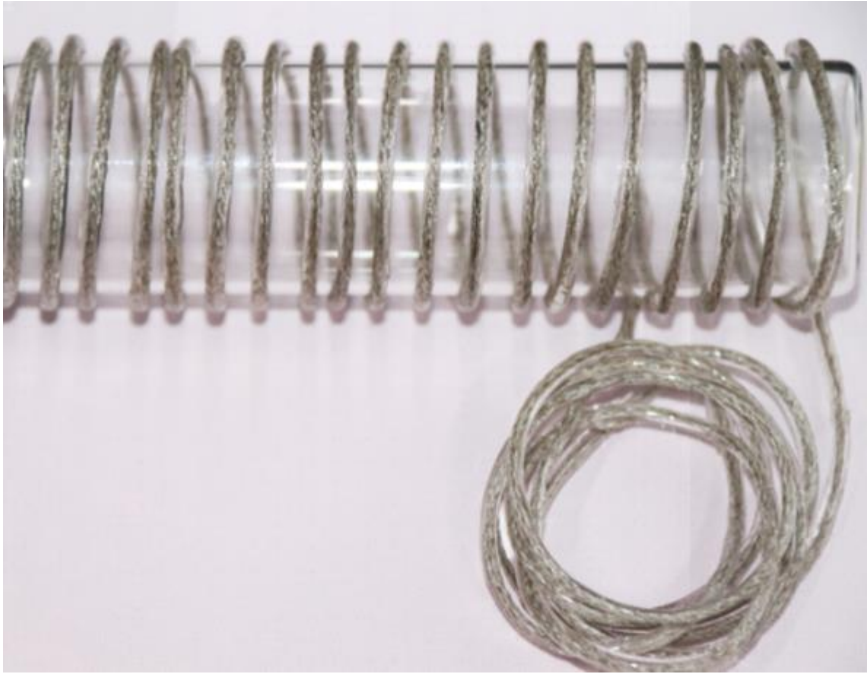

Supplementary Figure 9. **Photograph image of the continuous PDMS-coated energy yarn.** The PDMS-coated energy yarn with eight-axial yarn electrode and 2 mm diameter was chosen as the braided yarn to fabricate the 3DB-TENG.

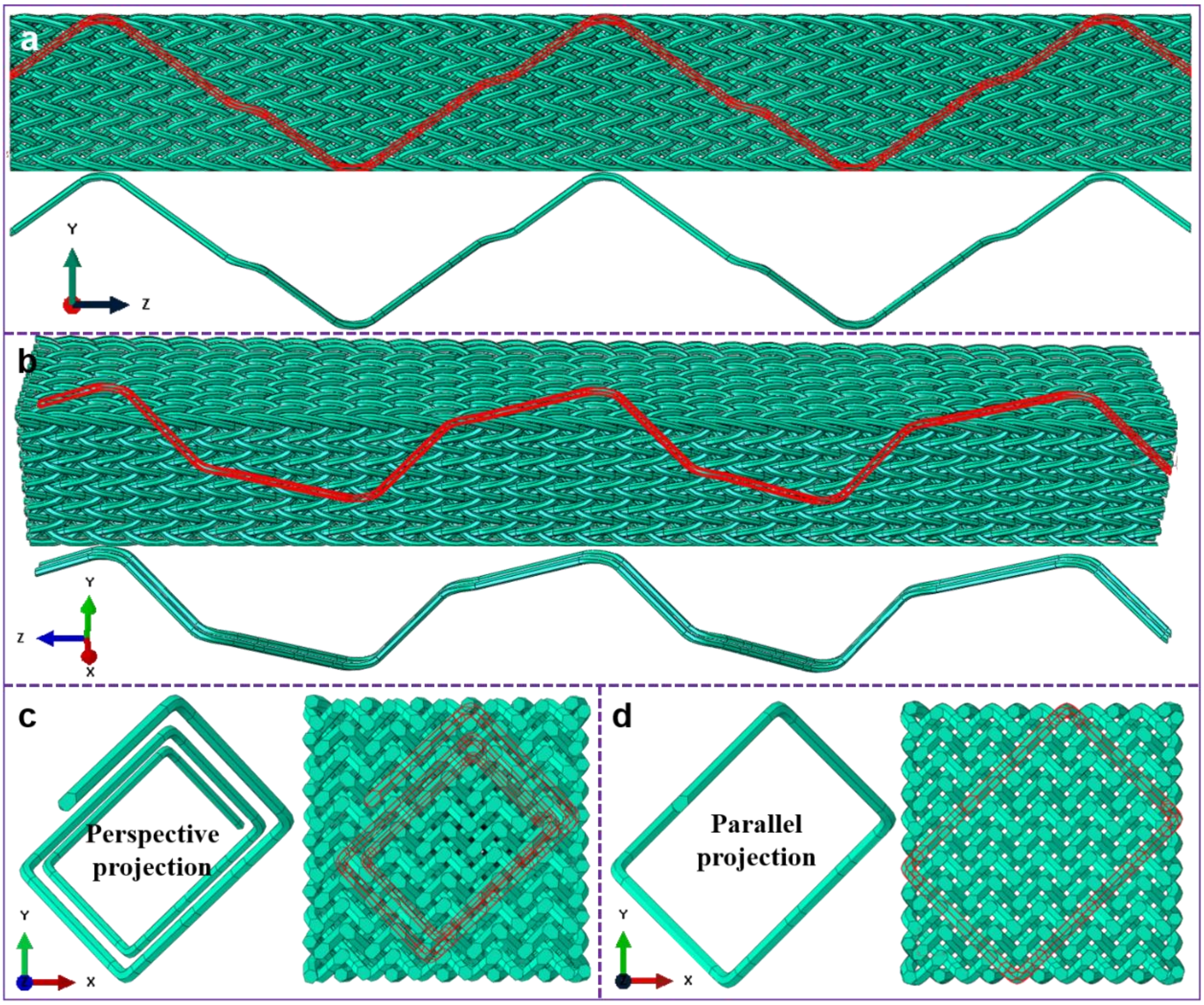

Supplementary Figure 10. **The spatial motion trajectory of a single braided yarn in the 3D braided fabric. a** Lateral view in the YOZ plane. **b** 3D view. **c, d** Vertical view in the XOY plane from the visual angle of (**c**) perspective and (**d**) parallel projections.

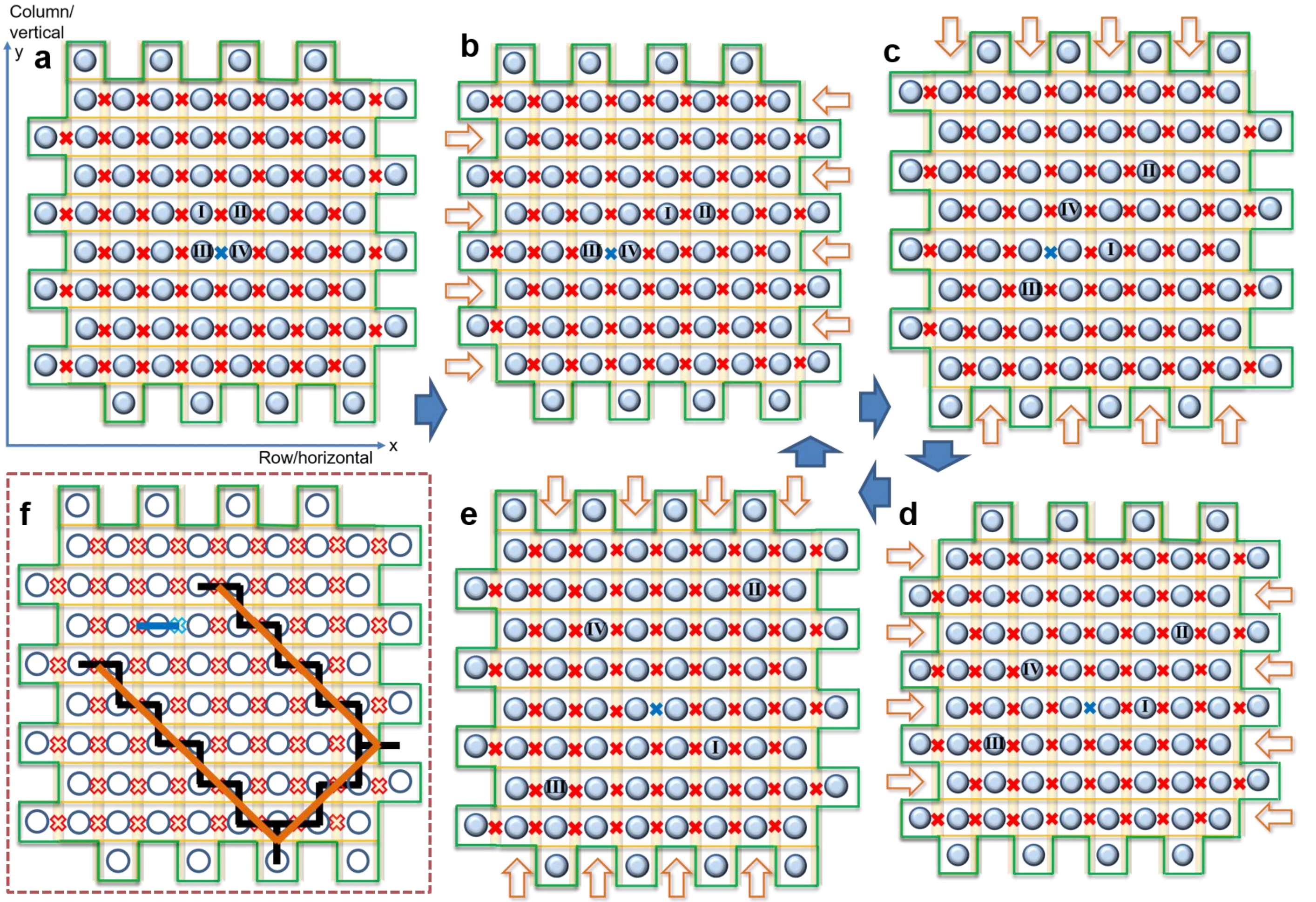

Supplementary Figure 11. **Schematic illustration of the four-step rectangular braiding process. a** Distribution of yarn carriers on the machine bed in the initial state. **b** The first braiding step. **c** The second braiding step. **d** The third braiding step. **e** The fourth braiding step. **f** Demonstration of the motion trajectories of the braiding yarn, the axial yarn, and their yarn carriers. The moving routes of one braiding yarn and its connected yarn carrier are marked with orange and black lines, respectively. Meanwhile, the motion track of the axial yarn and its carrier are the same, which is indicated by blue line.

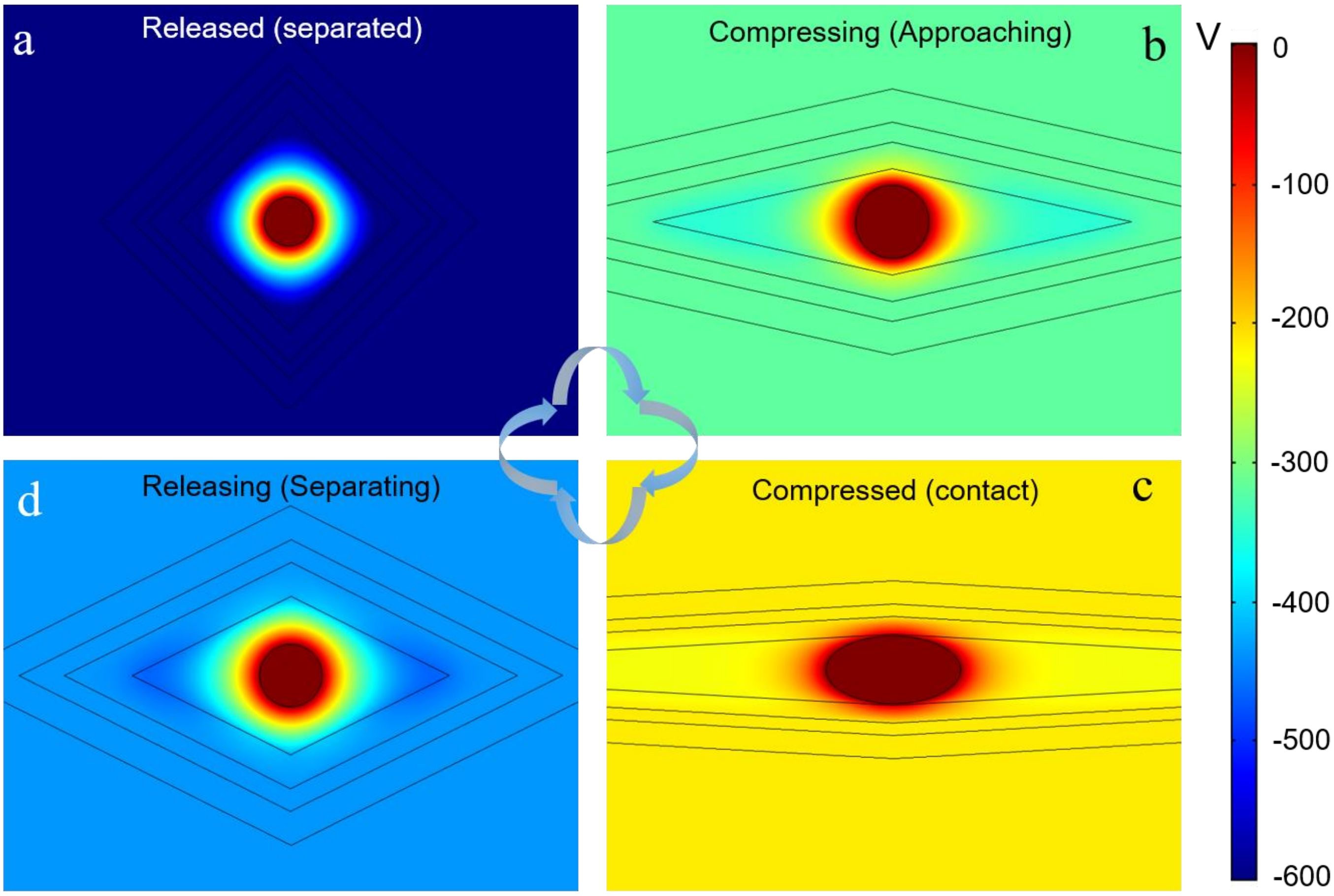

Supplementary Figure 12. Electrical potential distribution of the 3DB-TENG during one contact and separation process. The electrical potential distribution is simulated by the finite element software of COMSOL Multiphysics. **a-d** A complete contact-separation process mainly consists of four states, including (**a**) fully separated. (**b**) gradually approaching to, (**c**) fully contact (or maximum compression state), and (**d**) gradually separating away.

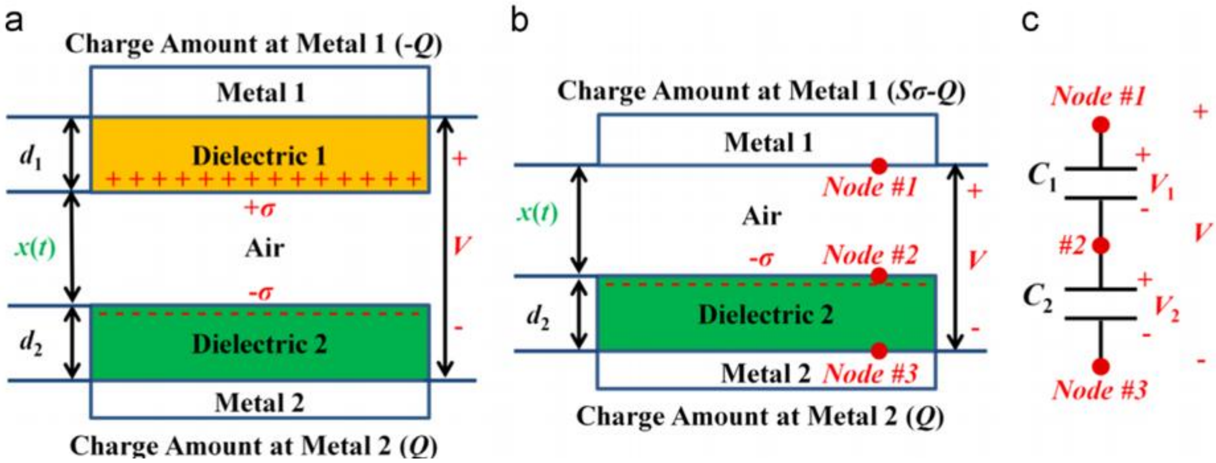

Supplementary Figure 13. Theoretical models for the attach-electrode parallel-plate contact-mode TENG. **a** dielectric-to-dielectric attached-electrode parallel-plate contact-mode TENG. **b** Conductor-to-dielectric attached-electrode parallel-plate contact-mode TENG. **c** Equivalent circuit diagram for conductor-to-dielectric attach-electrode parallel-plate contact-mode TENG [1].

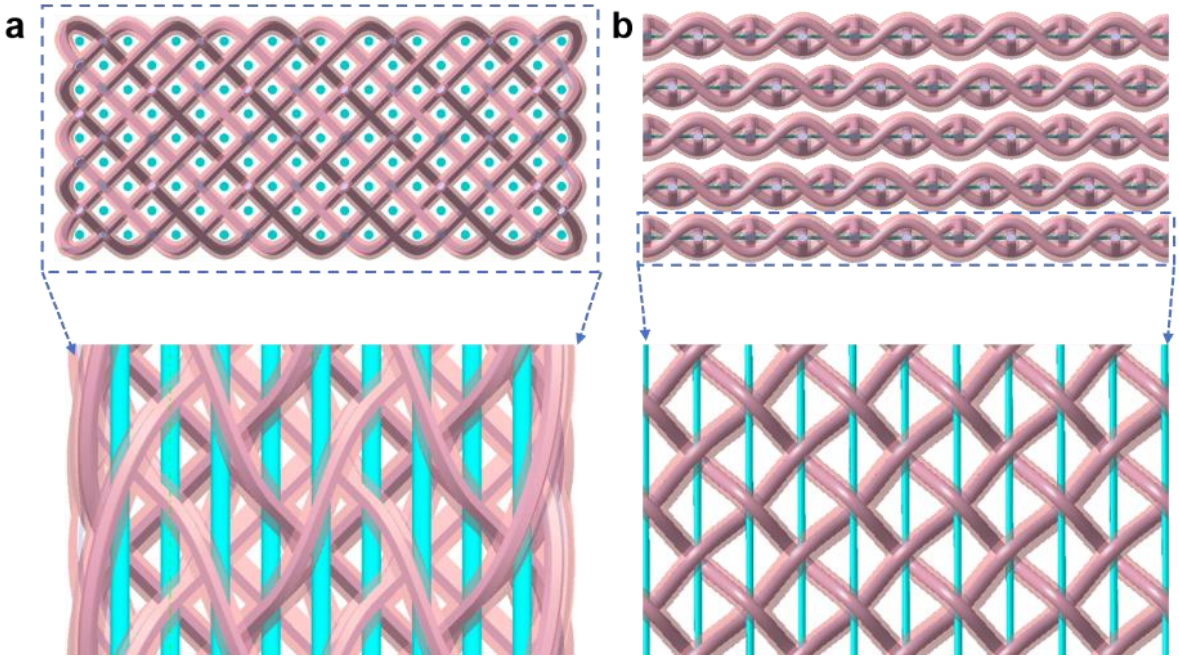

Supplementary Figure 14. Schematic illustration of the 3DB-TENG and the multilayered 2D triaxial braiding TENG fabric. a-b the cross sections (top) and surfaces (bottom) of (**a**) the rectangle-shaped 3DB-TENG and (**b**) the multilayered 2D triaxial braided TENG fabric. The length and width of the 3D and 2D TENG fabrics are tailored into the same size. By stacking 2D TENG fabrics along the thickness direction, the overall thickness of the multilayered 2D triaxial braided TENG fabric is approximately equal to that of 3DB-TENG.

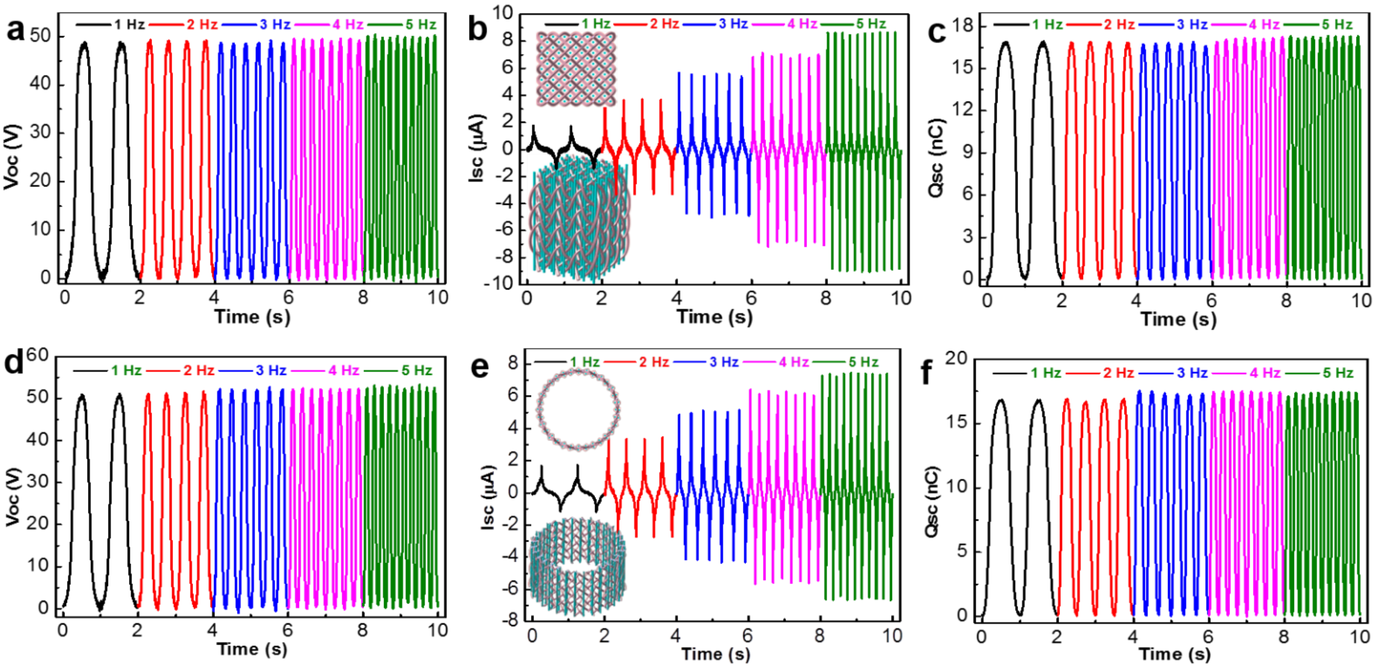

Supplementary Figure 15. Electrical output performance of the square-shaped and toroidal-shaped 3DB-TENGs. a-c Electrical outputs of the square-shaped 3DB-TENGs under different loading frequencies, including (**a**) V_{OC}, (**b**) I_{SC}, and (**c**) Q_{SC}. **d-f** Electrical outputs of the toroidal-shaped 3DB-TENGs under different loading frequencies, including (**d**) V_{OC}, (**e**) I_{SC}, and (**f**) Q_{SC}.

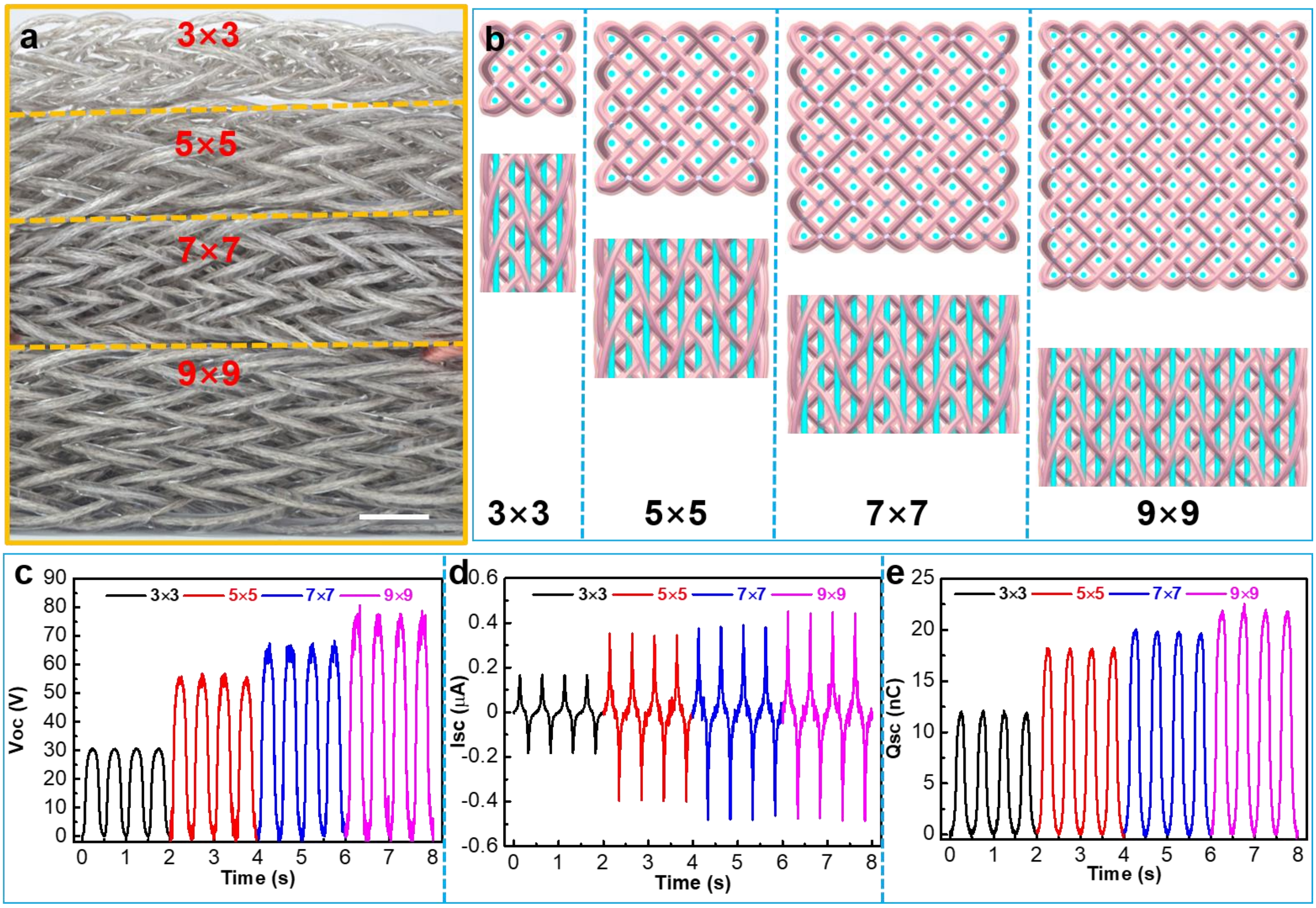

Supplementary Figure 16. Effect of the number of braiding yarns on the electrical output performance of the 3DB-TENG. a Photographs of the 3DB-TENGs fabricated from different numbers of braiding yarns (3×3, 5×5, 7×7 and 9×9, scale bar: 1 mm). **b** Schematic illustration of the 3DB-TENGs with different numbers of braiding yarns. The top is a view of the cross section while the bottom is a view of the surface morphology. **c-e** Electrical outputs of the 3DB-TENGs with different numbers of braiding yarns, including (**c**) V_{OC}, (**d**) I_{SC}, and (**e**) Q_{SC}.

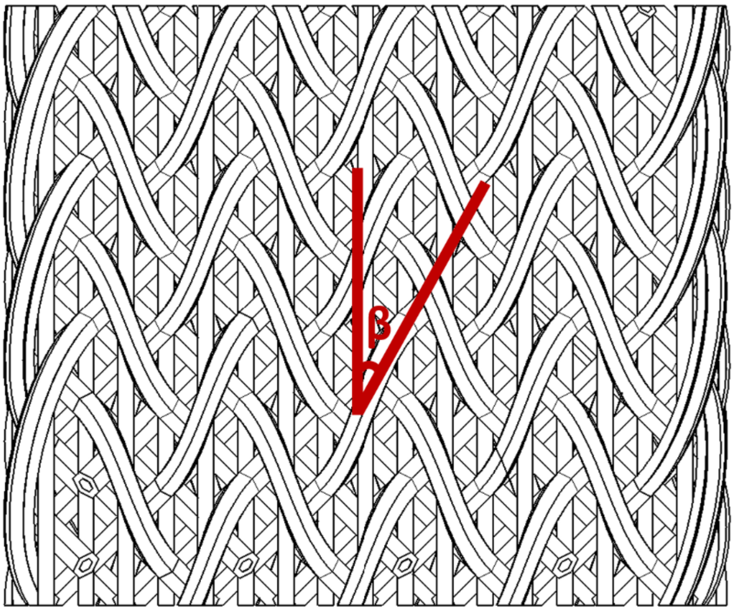

Supplementary Figure 17. Schematic illustration of the surface braiding angle (β) of the 3DB-TENG. The surface braiding angle (β) is defined as the angle between the axis of the surface braiding yarn and the Z-axis.

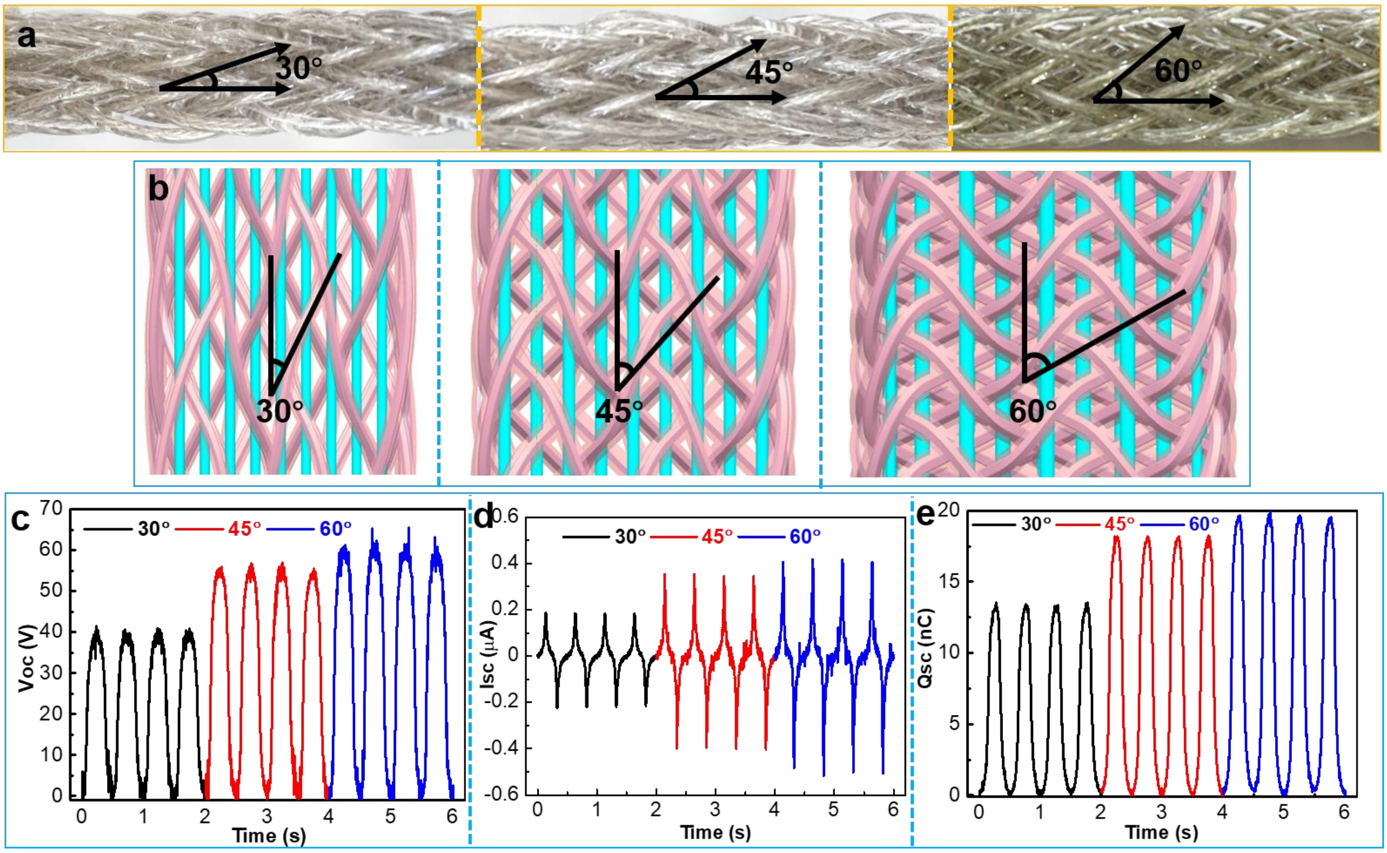

Supplementary Figure 18. Effect of the surface braiding angles (30°, 45° and 60°) on the electrical output performance of the 3DB-TENG. a Photograph images of the 3DB-TENGs fabricated with different surface braiding angles. **b** Illustration of the surface structures of the 3DB-TENGs with different surface braiding angles. **c-e** Electrical outputs of the 3DB-TENGs under different surface braiding angles, including (**c**) V_{OC}, (**d**) I_{SC}, and (**e**) Q_{SC}.

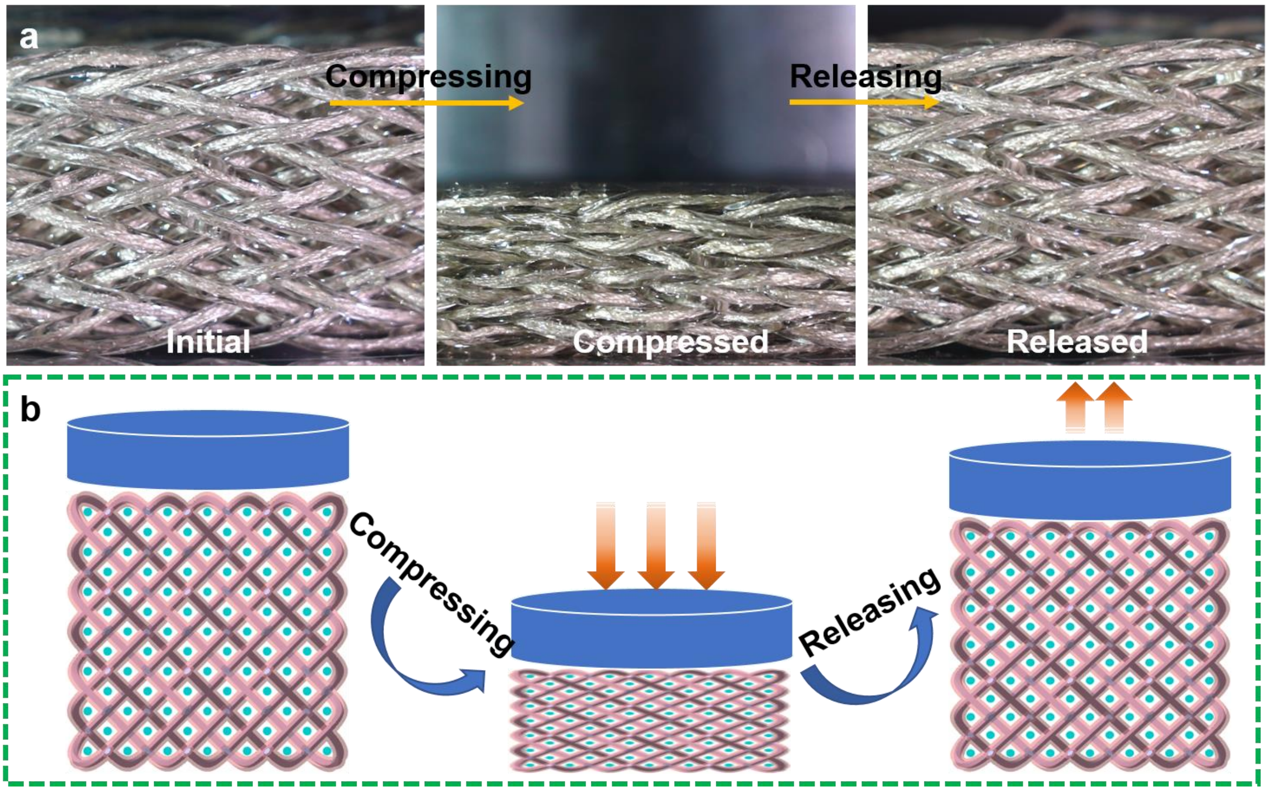

Supplementary Figure 19. Analysis of the compression resilience of the 3DB-TENG. a Photograph images of the 3DB-TENG under the loading-holding-unloading process. **b** schematic illustrations of compression behaviors of the 3DB-TENG under the loading-holding-unloading process.

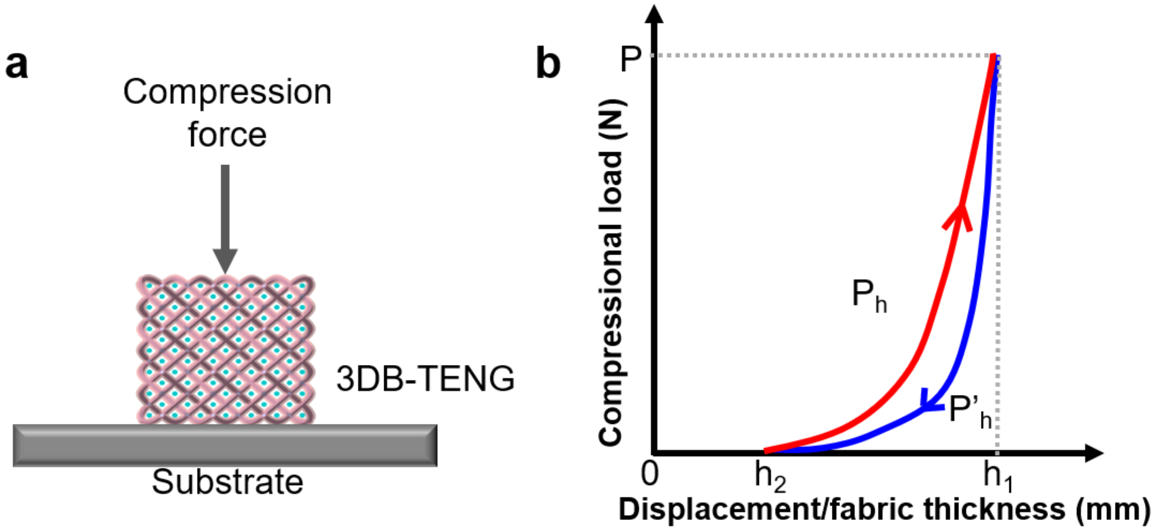

Supplementary Figure 20. Compression resilient curve of fabrics. a Schematic illustration of the compression process of the 3DB-TENG. **b** A typical pressure-displacement curve from the compression-recovery testing.

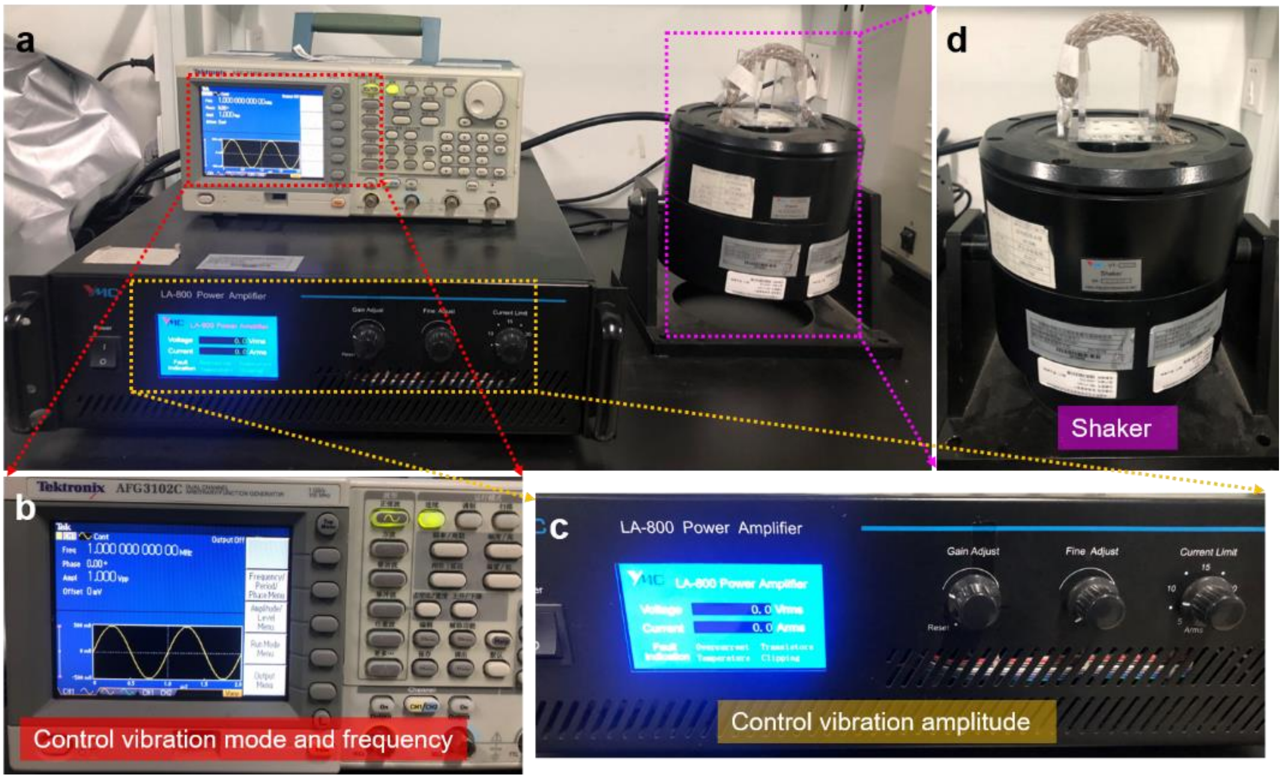

Supplementary Figure 21. Vibrational energy harvesting equipment of our 3DB-TENG. a Vibration acceleration testing platform. **b** A waveform controller (Tektronix AFG3102C). **c** A power amplifier (YMC LA-800). **d** An electrodynamic shaker (Labworks ET-126 shaker).

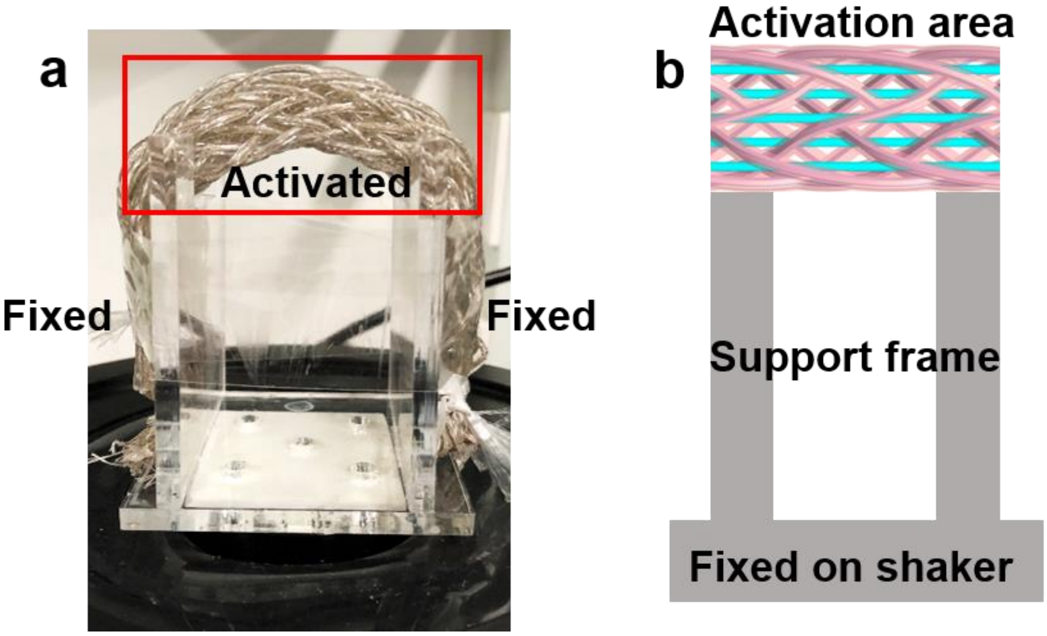

Supplementary Figure 22. Actual activation area of the 3DB-TENG for vibrational energy harvesting. **a** Photograph image of the 3DB-TENG sample fixed method on shaker. **b** Schematic illustration of the 3DB-TENG sample fixed method on shaker.

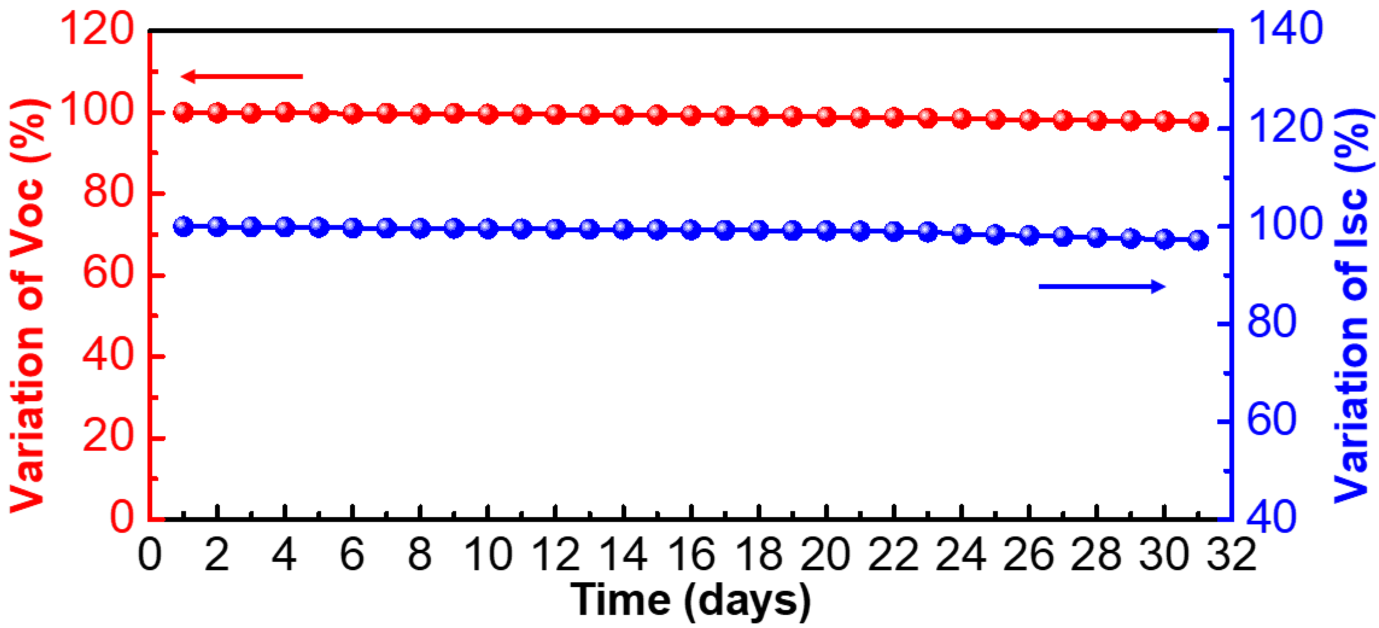

Supplementary Figure 23. Percentage change of electrical output of the 3DB-TENG under long-term working condition. The variations of the open-circuit voltage (V_{OC}) and short-circuit current (I_{SC}) of the 3DB-TENG are measured during one month.

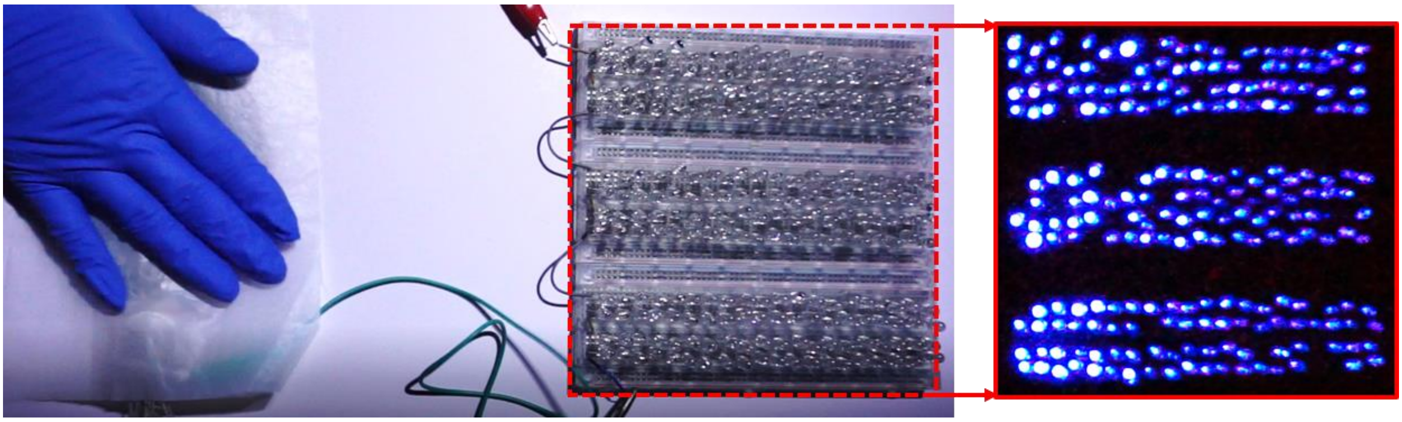

Supplementary Figure 24. Demonstration of the power generation capability of the 3DB-TENG. It can be found that the 3DB-TENG can light up hundreds of LEDs just by hand tapping.

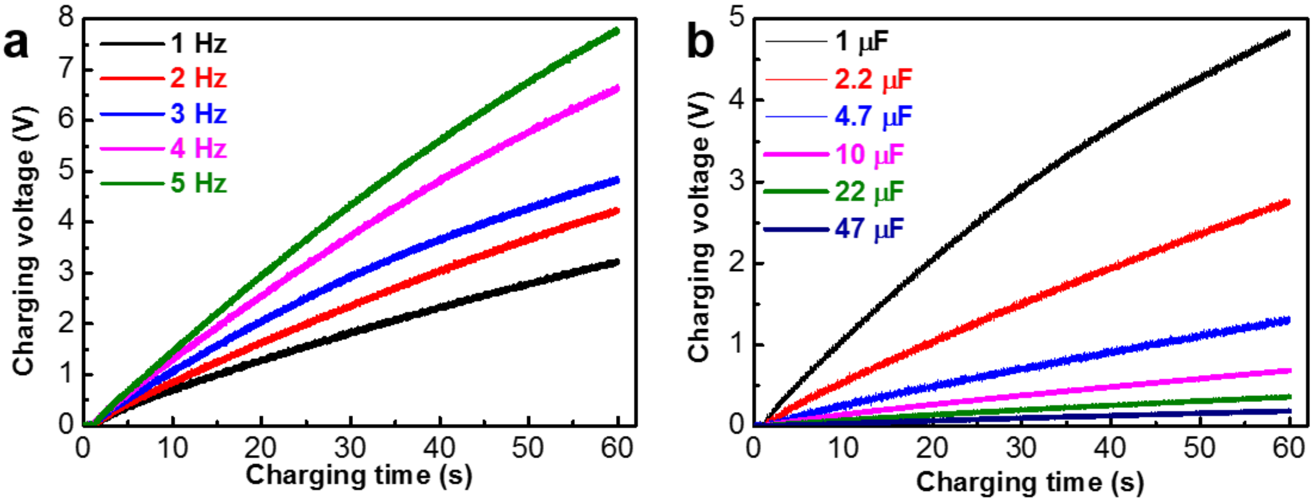

Supplementary Figure 25. Charging abilities and characteristics of the 3DB-TENG. a Charging curves under different loading frequencies. **b** Charging curves under different capacitor capacities.

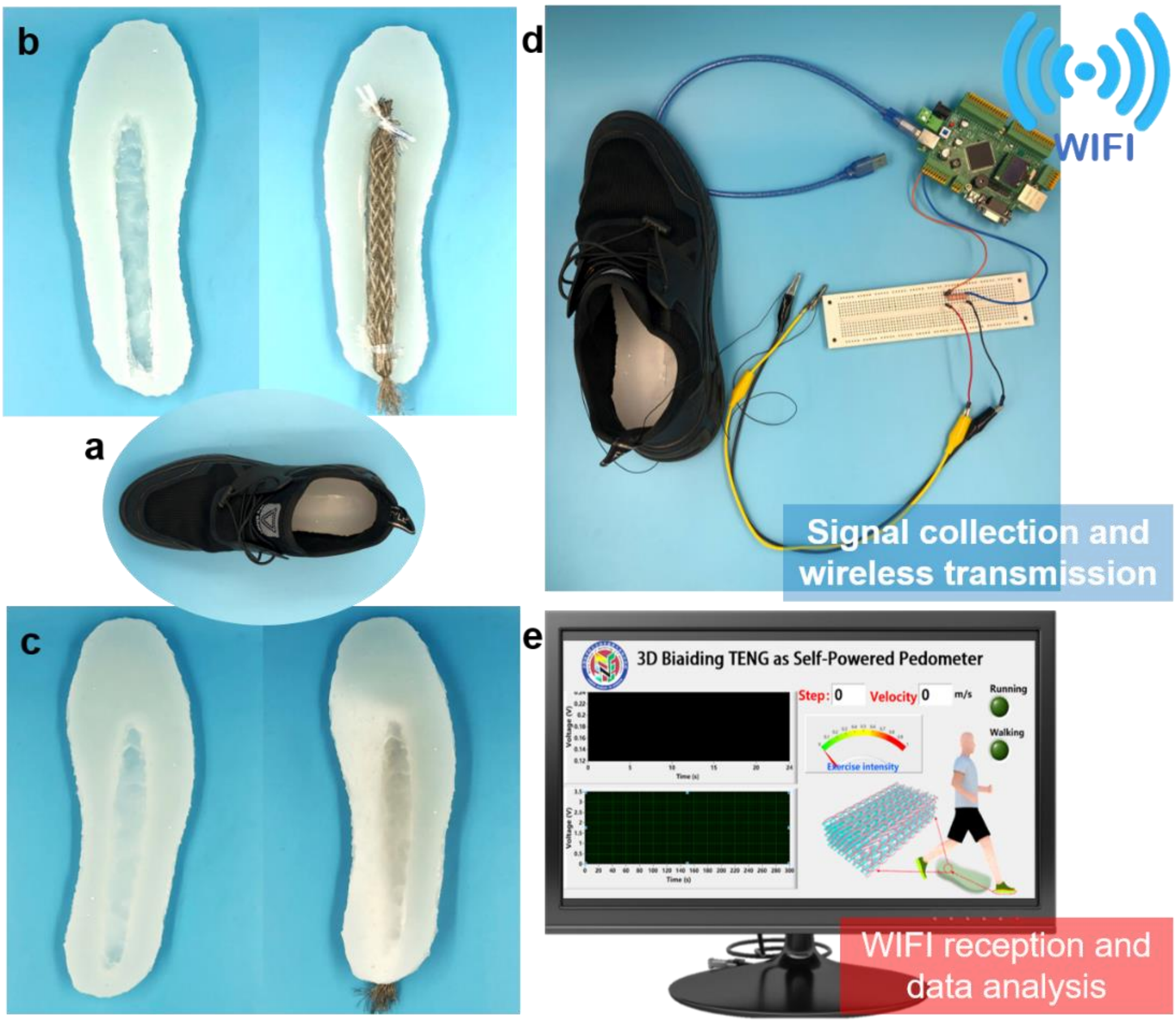

Supplementary Figure 26. Photograph image of actual integration of the intelligent footwear system. a A smart shoe in concept with an energy harvesting sole. **b** the obverse and **c** the reverse of an energy harvesting and signal transmitting sole. The energy harvesting and signal transmitting sole includes a 3D braiding TENG devices in the middle and a surround silicone rubber packaging layer. **d** Circuit connection of the signal collection and wireless transmission system. **e** Output platform for WIFI reception and data analysis.

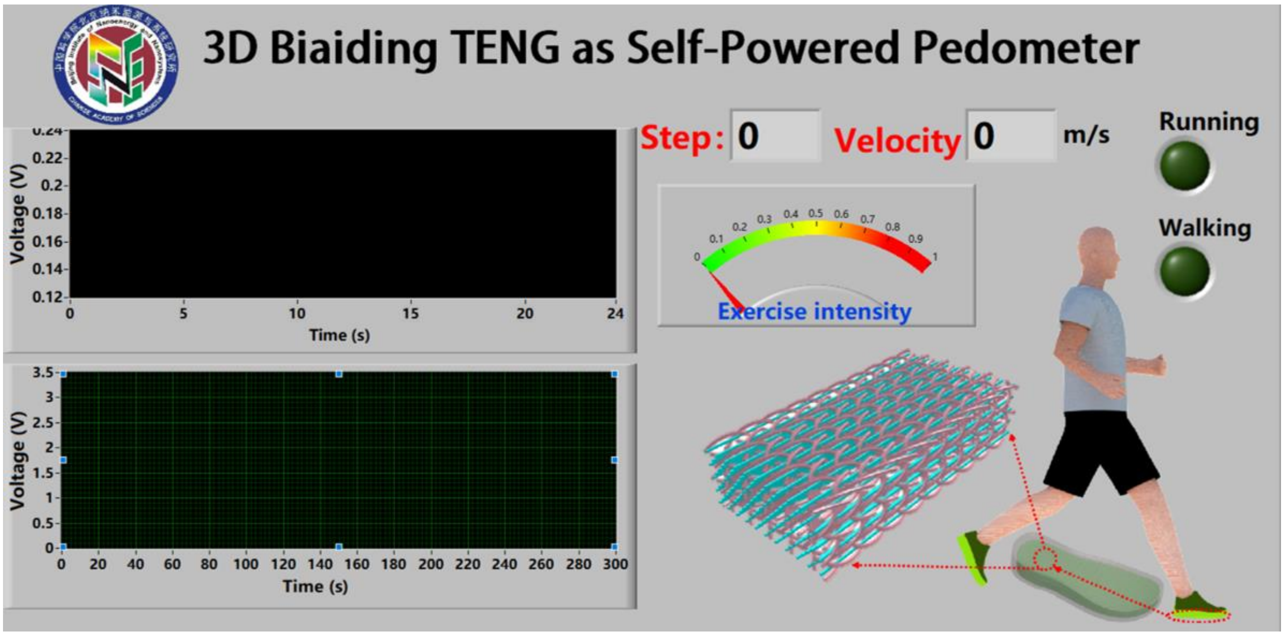

Supplementary Figure 27. Output interface for the demonstration of the intelligent footwear system. The developed output interface includes a real-time voltage signal display window, real-time motion data display window (including step, velocity and exercise intensity), and a switchable activation state between walking and running.

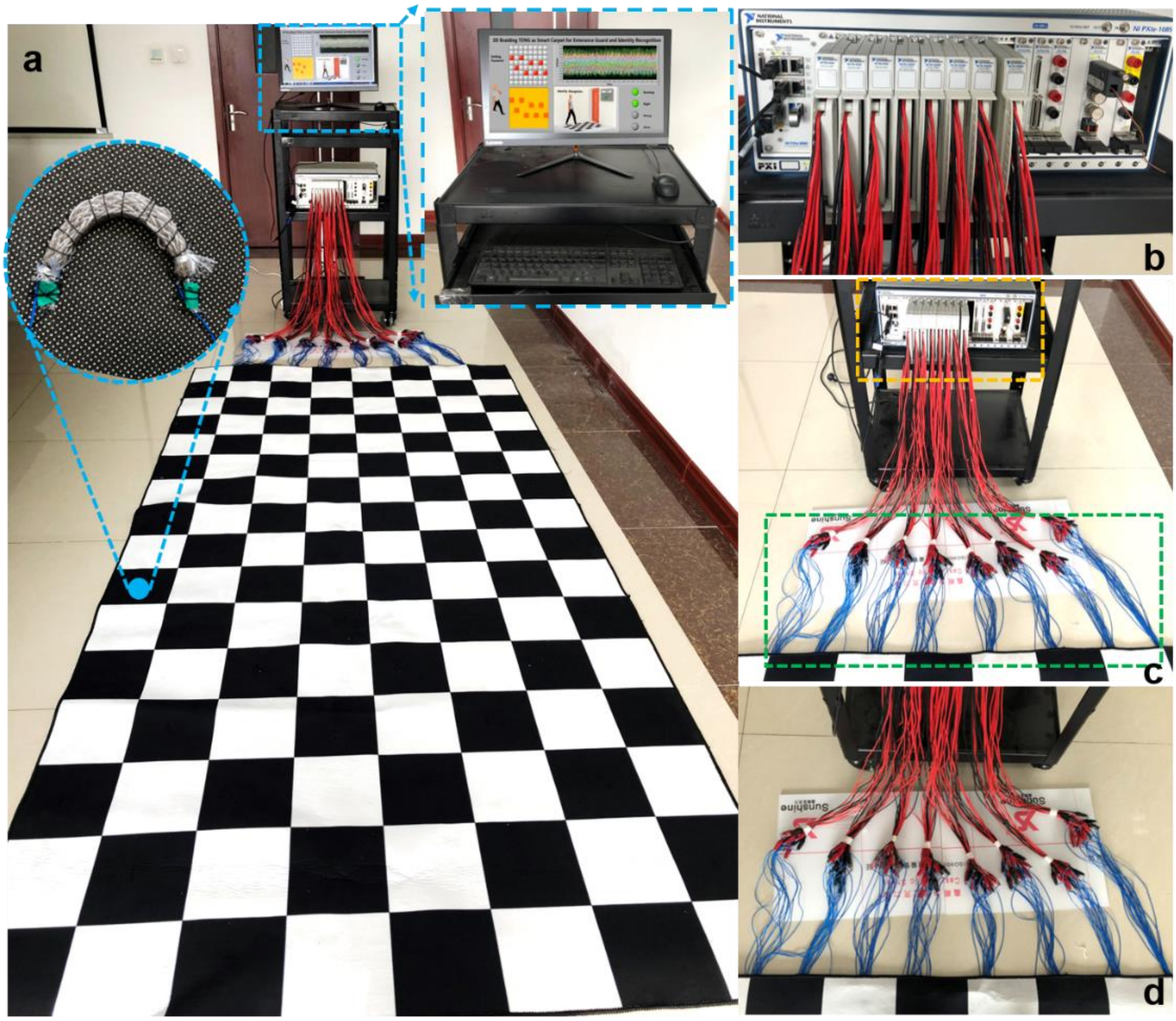

Supplementary Figure 28. Photograph of the actual integration system of the self-powered identity recognition carpet. a Holistic photograph image of the actual integration of the self-powered identity recognition carpet system. **b** Enlarged view of multi-channel (64) data acquisition system (PXIe-4300, National Instruments). **c** Integration system. **d** Circuit connection mode for the carpet.

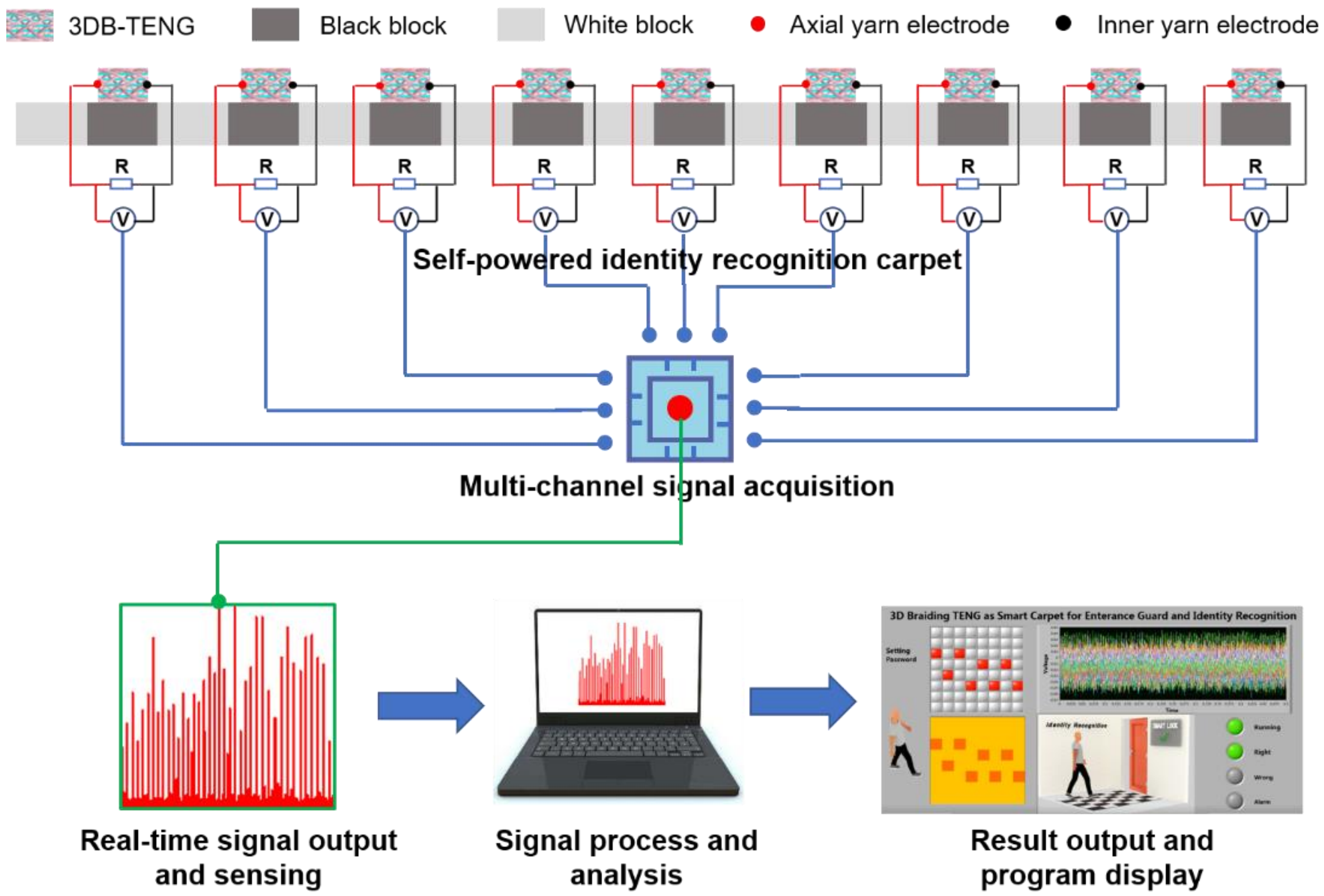

Supplementary Figure 29. The flowchart of the actual integrated system for the self-powered identity recognition carpet. The actual integrated system for the self-powered identity recognition carpet includes multi-channel voltage signal acquisition, synchronous data processing and analysis, real-time result output and display.

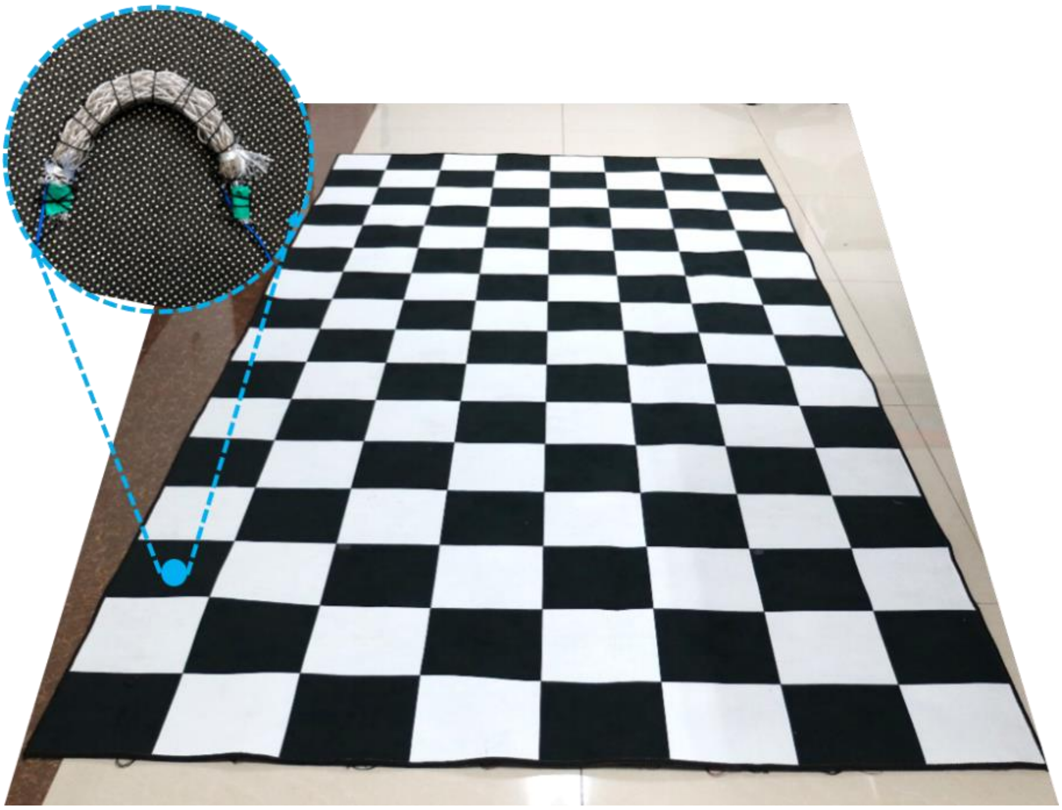

Supplementary Figure 30. Photograph of the smart carpet for safeguarding entrances and identity recognition. The self-powered identity recognition carpet is made up of 128 equally-sized black and white checkered square blocks. The size of each block is fixed as 20×20 cm^2. The 3DB-TENG fabrics are sewn in horseshoe shape at the back center of the black blocks.

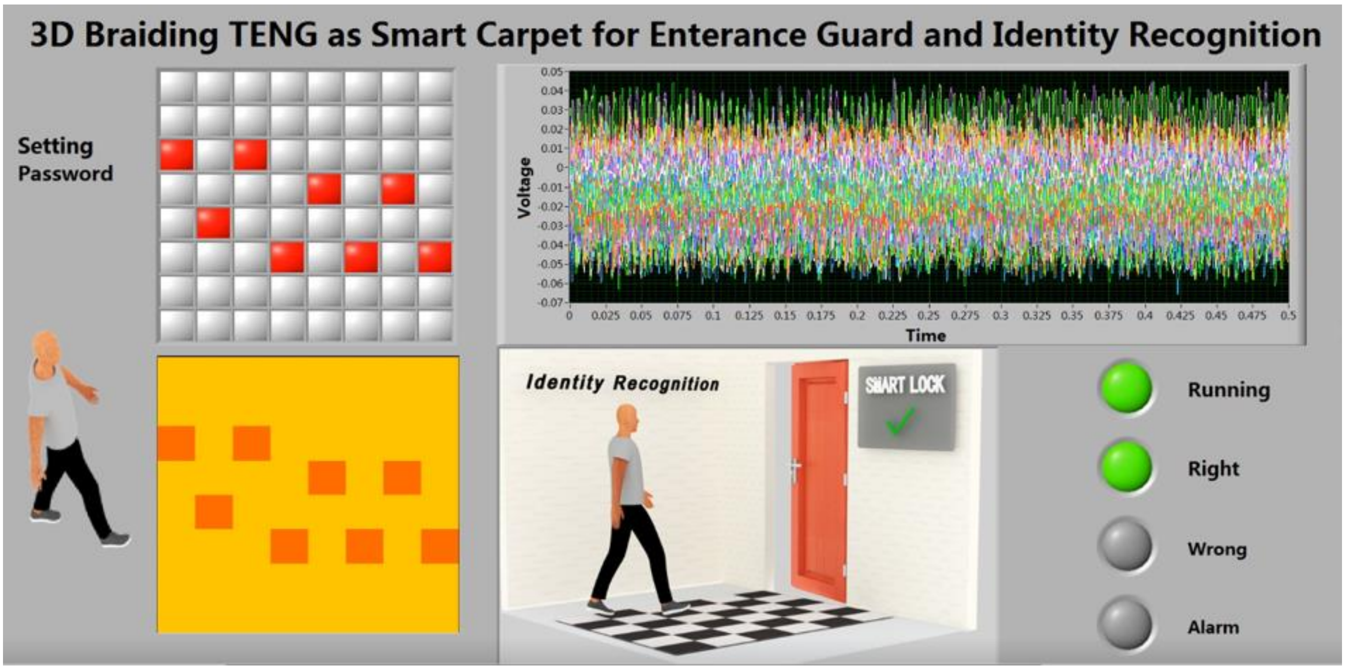

Supplementary Figure 31. The developed output interface of the smart carpet system. The output interface includes the real-time displayed walking path, set password path, the real-time voltage output signal of the 64 sensing units, and switchable judgment states.

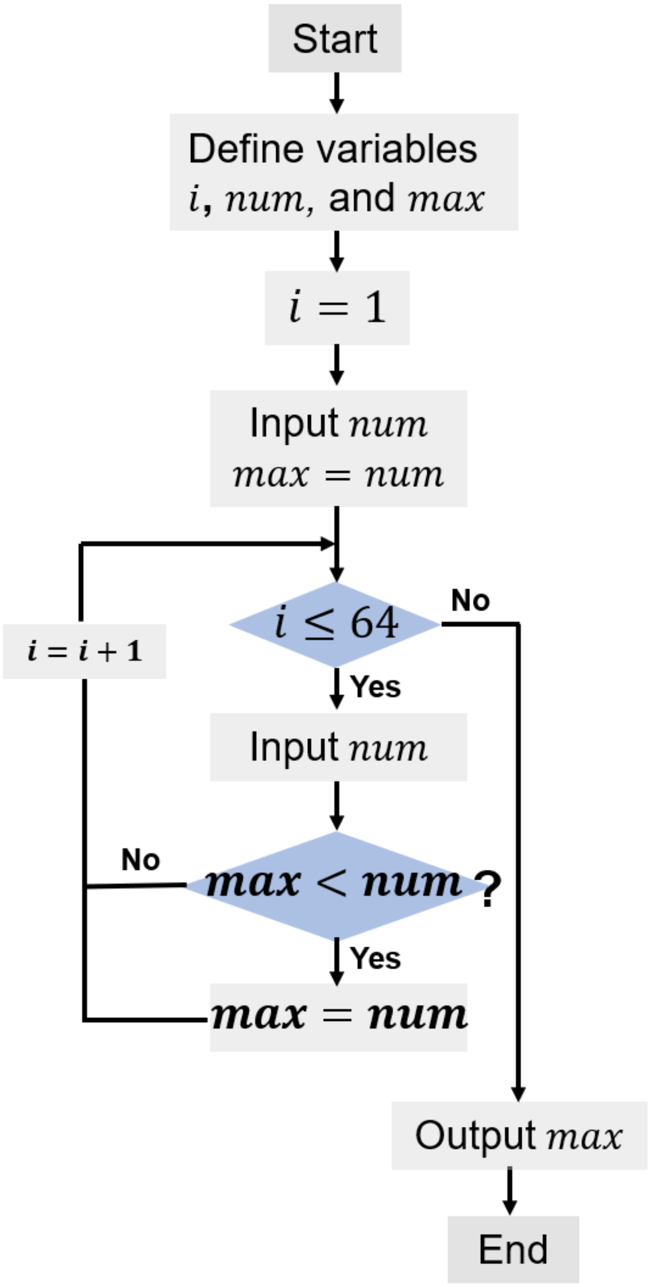

Supplementary Figure 32. Algorithm flowchart of the self-powered identity recognition carpet. The algorithm flowchart can distinguish the location of the contact area quickly and accurately, which can search the maximum value among the 64 voltage signals.

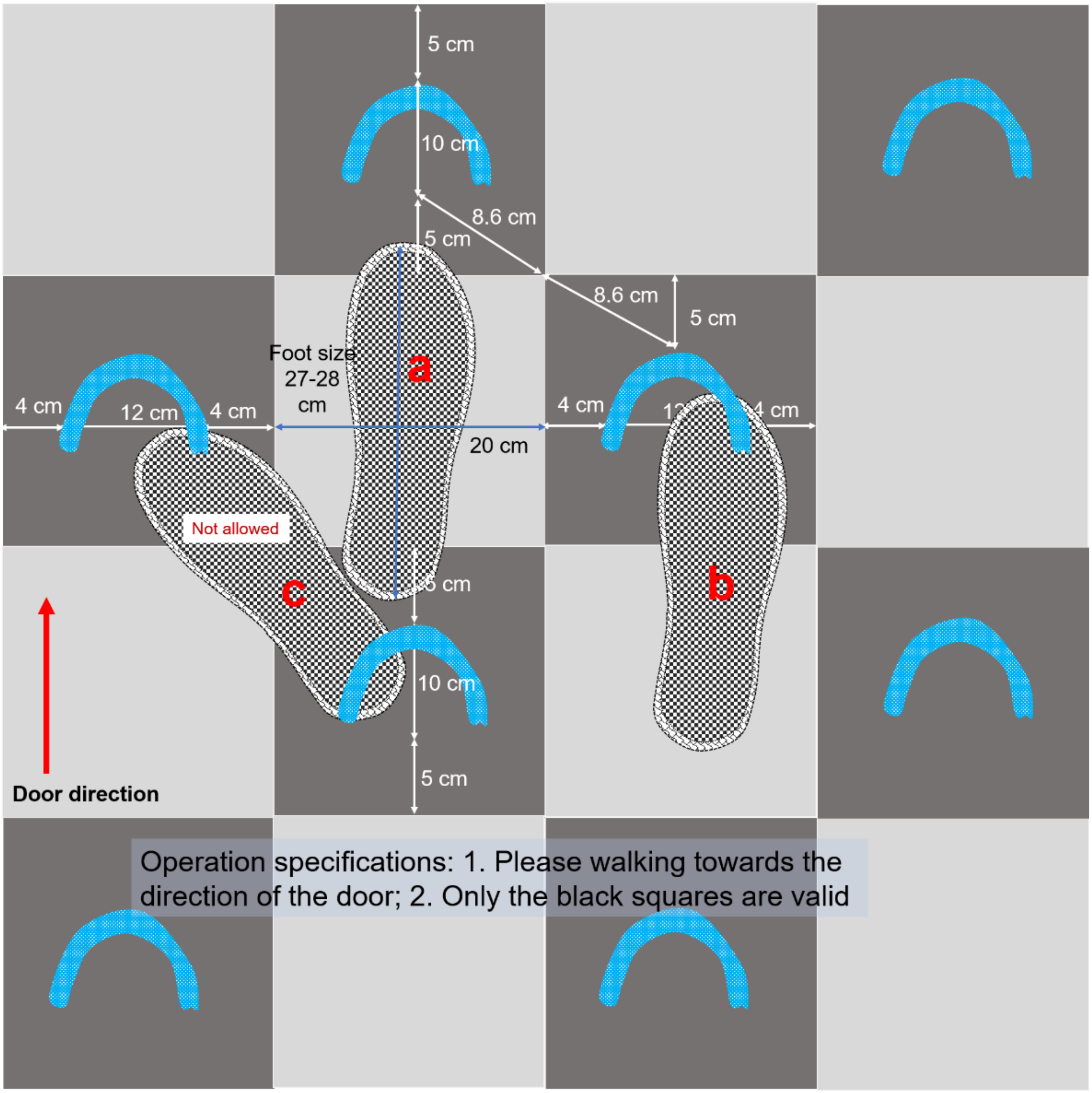

Supplementary Figure 33. The locations of our 3DB-TENGs in the self-powered identity recognition carpet. The soles marked with **a**, **b** and **c** represent stepping on no, one and two sensing areas, respectively. The 3DB-TENGs are sewn in a horseshoe shape on the back of the carpet sections with black blocks.

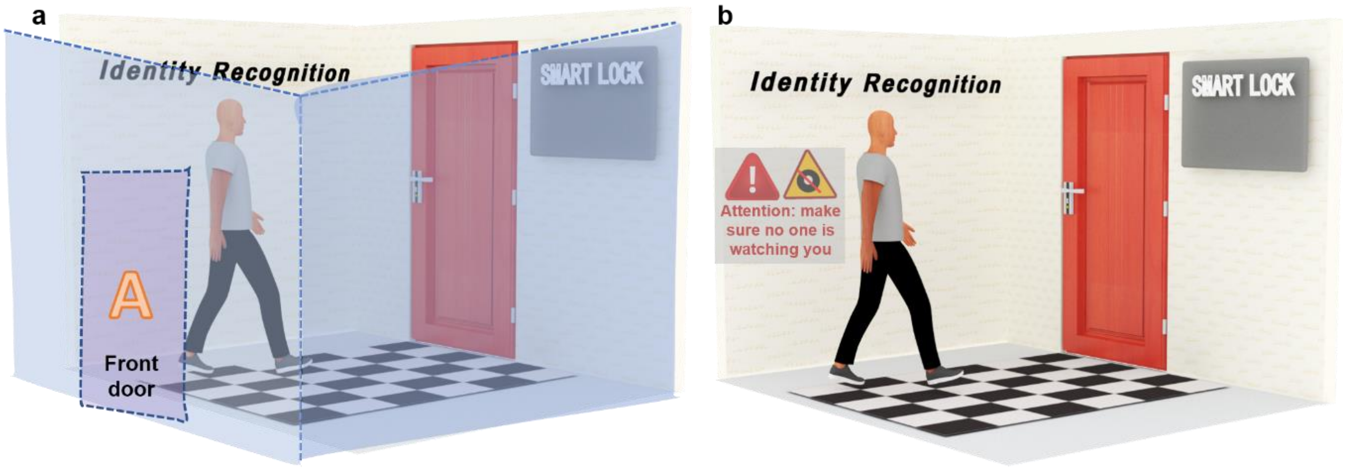

Supplementary Figure 34. The potential measures to eliminate or prevent potential peeping behaviors as much as possible. a Adding an isolation room where there is the identity recognition carpet, just like the arrangement of the bank's automatic teller machine (ATM). **b** Adding warning signs (Attention or no peeping) and information (e.g. "Make sure no one is watching you") at the entrance.

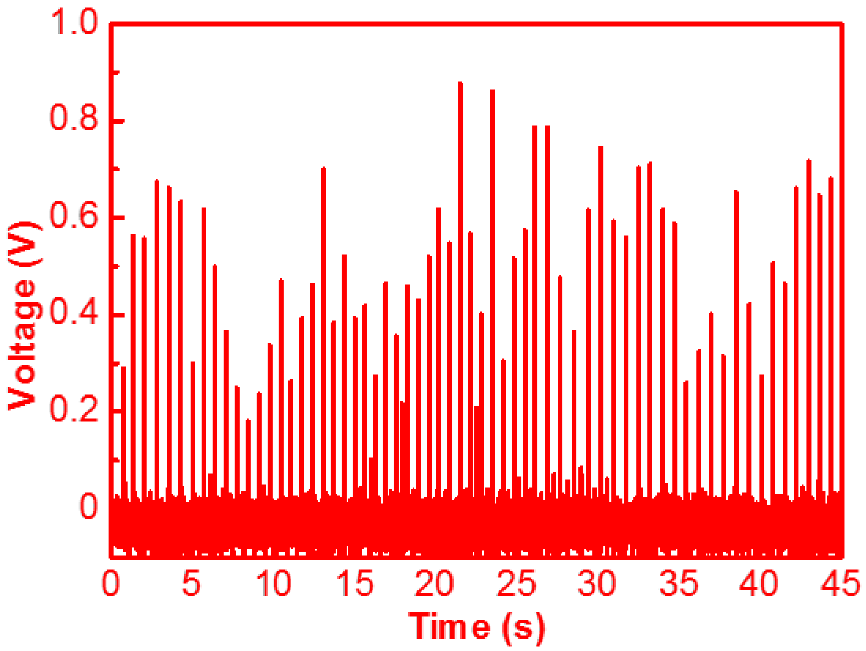

Supplementary Figure 35. Continuous voltage output signals of one sensing unit (black block) on the self-powered identity recognition carpet. It can be found that the voltage output signal has good stability and reliability.

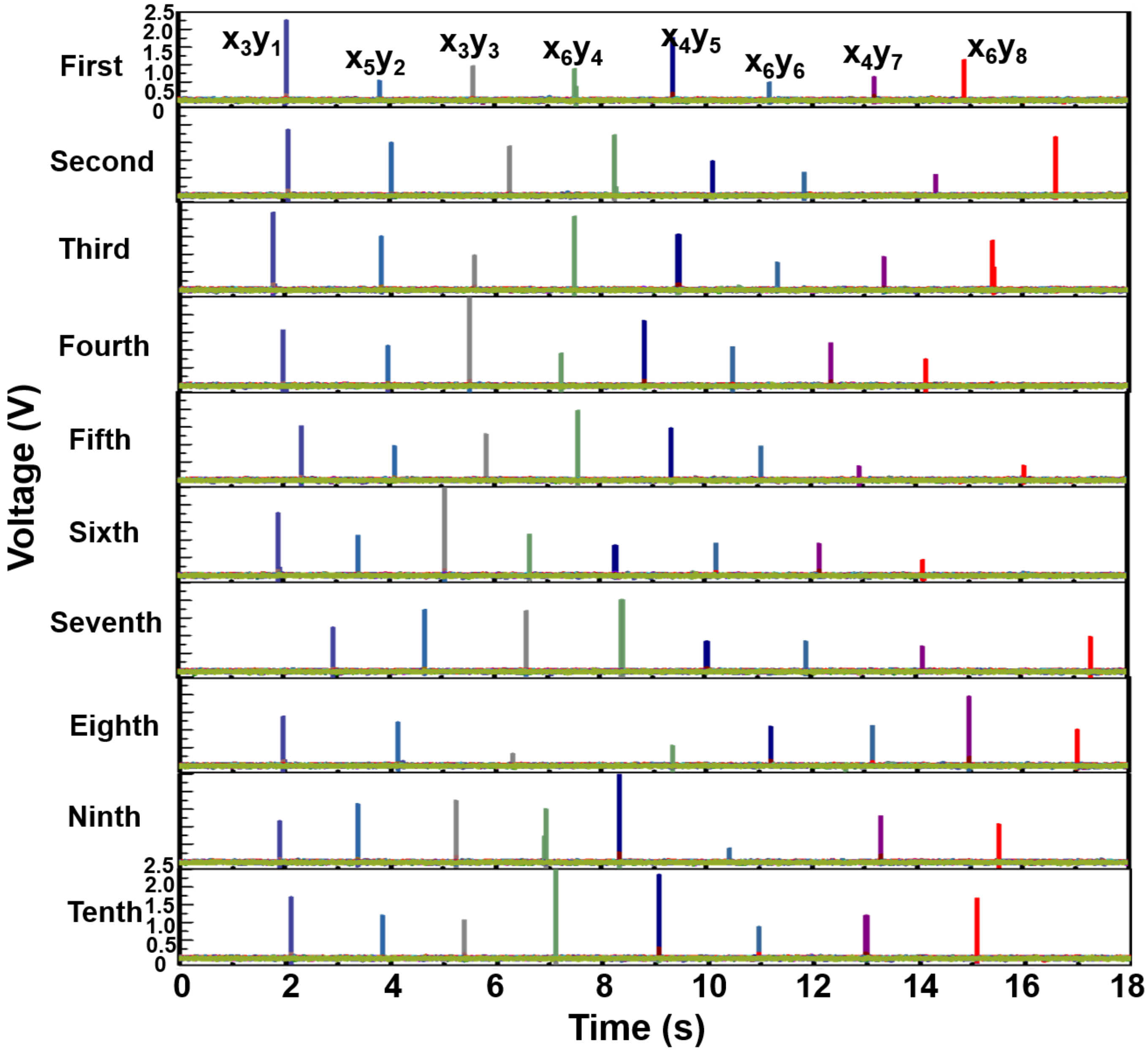

Supplementary Figure 36. Repeatability and reliability of the self-powered identity recognition carpet. The voltage output signals of the smart carpet obtained by continuously walking along the correct password path for ten times are compared, which show good repeatability and reliability.

Supplementary Notes

Supplementary Note 1. Coordination and balance of increasing electrical output and reducing winding yarn numbers in textile manufacturing

On one hand, our experimental results show that the electrical conductivity and tensile strength of winding yarns, and the electrical output performance of energy yarns will slightly increase with the increasing of the number of winding yarns. On the other hand, the increase of the number of winding yarns will inevitably lead to the increasing of manufacturing process and production cost, and even cause the compatibility issue of machines. However, we think that we can well deal with or balance this selection dilemma after we fully consider and weight the following factors, such as the performance requirements of terminal products, the potential application occasions, the operation capability of existing machines, and the selection of target consumer groups. Among them, the performance requirements of terminal products include yarn diameter, mechanical strength, electrical conductivity, electrical output capability, and etc. For example, if we want to prepare energy yarns with high conductivity and high tensile property, it is necessary to increase the number of winding yarns. In addition, the operation capability of existing equipment is also an important factor. For example, the size limitations of modern loom, such as the diameter of heddle hole, the spacing of reed, and the density of fabric, are difficult to meet the production requirements of the yarns with large diameters. Moreover, potential application occasions and target consumer groups are directly related to the final product quality and prime cost input, which in turn involves the selection of yarn numbers.

Generally, in order to balance the two aspects, we should comprehensively consider many factors, and make corresponding changes according to the actual needs. There is no general conclusion on this issue. However, in our personal opinion, considering the electrical output performance of energy yarn is not significantly enhanced as the number of winding yarns increases, we suggest that a smaller number of yarns should be preferred for actual textile manufacturing.

Supplementary Note 2. Four-step rectangular braiding process

More attention should be given to the track movements of the braiding and axial yarn carriers during one machine cycle in order to fully explain the process. At the first braiding step, the braiding yarn carriers and the axial yarn carriers move horizontally one position in an alternating manner. At the second step, the braiding yarn carriers move vertically one position in an alternate manner, while all the axial yarn carriers do not move and stay in their current positions. The third step and the fourth step reverse the carrier movements in the first and second steps, respectively. After these four steps, all the yarn carriers on the machine bed return to the original pattern, completing one machine cycle. A certain jamming action is imposed on all the yarns, which makes the yarns more or less closely intertwined. It is noteworthy that although the overall layout of yarn carriers on the machine bed is consistent with the original state, the actual positions of the individual yarn carriers have moved. As shown in Supplementary Fig. 11, the four braiding yarn carriers (I, II, III and IV) are in close proximity in (a), which will be changed to far away after one four-step braiding process. The motion track of one braiding yarn carrier is outlined with a black line in (f), which follows a zig-zag trace through an alternating movement in rows and columns. The movement of the braiding yarn is always half a step behind the carriers. Supposing the arrangement of carrier on the machine bed is a reflection of the cross-section of the 3D braiding fabric, the carrier is the position control point of the yarn trajectory in plane. According to the least-squares principle, the trace of braiding yarn in the cross-section of fabric will be the connecting line of the mid-point of the carriers. Therefore, the movement route of braiding yarn can be found, as indicated with orange line in (f). The axial yarn carrier only moves along the X direction and returns to the original position after one machine cycle, as does the yarn, which is marked with a blue line in (f). Different from the braiding yarn, the axial yarn does not participate in braiding but only embeds evenly in between the braiding yarns.

Supplementary Note 3. The relationship between motion frequency and basic electrical output performance of TENGs

The models built for dielectric-to-dielectric and conductor-to-dielectric contact-mode TENGs are shown in Supplementary Fig. 13a and 13b, respectively. The V-Q-x relationship for the contact-mode TENGs can be derived based on electrodynamics. Therefore, the basic equation is given by [1]:

$$V = -\frac{Q}{S\varepsilon_0}\left(d_0 + x(t)\right) + \frac{\sigma x(t)}{\varepsilon_0} \tag{1}$$

The output voltage (V_{OC}) for an open circuit, the transferred charge (Q_{SC}), and the output current (I_{SC}) in short-circuit conditions are as follows [1]:

At open-circuit condition, there is no charge transfer, which means

$$V_{OC} = \frac{\sigma x(t)}{\varepsilon_0} \tag{2}$$

At short-circuit condition, V is 0. Therefore, the transferred charges are

$$Q_{SC} = \frac{S\sigma x(t)}{d_0 + x(t)} \tag{3}$$

$$I_{SC} = \frac{dQ_{SC}}{dt} = \frac{S\sigma d_0}{\left(d_0 + x(t)\right)^2}\frac{dx}{dt} = \frac{S\sigma d_0 v(t)}{\left(d_0 + x(t)\right)^2} \tag{4}$$

where S is the triboelectric surface area. $x(t)$ is the time-dependent distance between the two triboelectric layers. ε_0 is the permittivity of free space, σ is the tribo-charge surface density, and $v(t)$ is the speed of the relative mechanical movement. The effective dielectric thickness d_0 is defined as the summation of all the thickness of the dielectric d_i between the two metal electrodes divided by its relative effective thickness ε_{ri} (Supplementary Fig. 13c), as shown below:

$$d_0 = \sum_{i=1}^{n} \frac{d_i}{\varepsilon_{ri}} \tag{5}$$

From Supplementary Equation 2 and 4, it can be found that the V_{OC} and Q_{SC} are independent of speed, which means that the variation of movement frequency will cause no change of the V_{OC} and Q_{SC}. However, the I_{SC} depends on the relative movement speed, which presents a positive correlation feature with the increase of speed.

Supplementary Note 4. Comparison of the power output between the 3DB-TENG and the multilayered 2D braiding TENG fabric

Several 2D triaxial braiding TENG fabrics were fabricated with the same length and width as these of the 3D rectangle-shaped TENG fabric. Afterwards, several 2D fabric TENGs were stacked along their thickness direction until their overall thickness is approximately the same as that of the 3D fabric TENG, as shown in Supplementary Fig. 14. Finally, both of the electrodes coated with PDMS and the axial electrodes are connected in parallel. The higher electrical output capability of 3D TENG fabric can be attributed to its special internal structure, which provides more contact-separation space for the PDMS-coated energy yarns and the axial conductive yarns than that in the multilayered 2D TENG fabric.

Supplementary Note 5. Factors affecting the optimum external resistance of TENGs

The theoretical study of contact-mode TENGs in reference [1] shows that the optimum resistance of TENGs can be approximately given by the following equation:

$$R_{opt} = \frac{d_0^2}{F_{opt}^2 S v \varepsilon_0} \approx \frac{(d_0+x_{max})^2}{S v \varepsilon_0} \quad (6)$$

where x_{max} is the maximum separation distance between two triboelectric layers. S is the area size of the dielectrics. ε_0 is the permittivity of free space. v is the average velocity. The effective dielectric thickness d_0 is defined as the summation of all the thickness of the dielectric d_i between the two metal electrodes divided by its relative effective thickness ε_{ri}, as shown below:

$$d_0 = \frac{d_1}{\varepsilon_{r1}} + \frac{d_2}{\varepsilon_{r2}} \quad (7)$$

The optimized value of F is only a function of y, as shown below:

$$F_{opt} = H(y) \approx \frac{1}{1+y} \quad (8)$$

The thickness factor y is defined as below:

$$y = \frac{x_{max}}{d_0} \quad (9)$$

The Supplementary Equation 6 can be utilized to estimate the optimum resistance for a TENG. Although for different moving modes, the actual optimum resistance will somewhat deviate from this estimated value, it can serve as a good reference when a TENG is designed for driving a specified load with certain resistance, so that the actual power on the load can be maximized [1].

From the above analysis, it can be found that the optimized resistance (R_{opt}) of TENGs is mainly related to the dielectric thickness d_0, the effective contact area S, the maximum separation distance x_{max}, and the permittivity of air ε_0. It can be found that R_{opt} is inversely proportional to either the area size (S) or the average velocity (v) while F_{opt} is not dependent on neither of these two parameters. R_{opt} increases with either the effective dielectric thickness (d_0) or the gap distance (x_{max}).

Supplementary Note 6. Evaluation of the figure of merit of the 3DB-TENG

The performance figure of merit (FOM_P) of TENGs consists of a structural figure of merit (FOM_S) related to the design of TENGs and a material figure of merit (FOM_M) as the square of the surface charge density [2]. The FOM_S and FOM_P can be calculated by equations below:

$$\mathrm{FOM_S} = \frac{2\varepsilon_0}{\sigma^2}\frac{E_m}{Ax_{max}} \tag{10}$$

$$\mathrm{FOM_P} = \mathrm{FOM_S}\sigma^2 = 2\varepsilon_0\frac{E_m}{Ax_{max}} \tag{11}$$

where σ is surface charge density (σ^2 can be the FOM_M), ε_0 is the permittivity of the vacuum, x_{max} is the maximum displacement, A is the triboelectrification area.

At present, the commonly used indexes to evaluate the electrical output performance of TENGs include open-circuit voltage (V_{OC}), short-circuit current (I_{SC}), short-circuit charge transfer (Q_{SC}), charging capacity, energy conversion efficiency, and power density. Among them, power density is an essential indicator to measure the power generation capacity of TENGs, because electrical outputs strongly depend on the dimension or size of test samples. According to sample dimensions, power output per unit scale can be divided into W/m (one dimension), W/m^2 (two dimension), and W/m^3 (three dimension). For TENGs with obvious three-dimensional (3D) structure (e.g. our 3DB-TENG), W/m^3 is the best indicator to characterize the power output density.

In addition, the power output density represented by W/m^3 has been widely used to evaluate the power output of TENGs with 3D structure [3,4]. Moreover, a TENG-based safety helmet (a power density up to 167.22 W/m^3) [5], a nanoparticle-enhanced TENG (a volume power density of 54.268 W/m^3) [6], a paper-based TENG (968 W/m^3) [7], and a liquid-FEP-based U-tube TENG (power density of 2.04 W/m^3) [8], and a rotating-disk-based TENG (a volume power density of 55.7 W/m^3) [9] are also typical examples.

Therefore, W/m^3 is undoubtedly also an easily relatable figure of merit.

Supplementary Note 7. Analysis of the compressional resilience coefficient of our 3DB-TENG

Supplementary Fig. 20 is a typical pressure-displacement curve from the compression-recovery testing. The compression resilience of textiles can be evaluated from the compression and decompression (recovery) curves [10]. The energy required to compress a fabric during compressional process (WC) can be calculated by the following equation:

$$WC = \int_{h_1}^{h_2} P_h dh \tag{12}$$

where P_h is the compressional force at fabric thickness h. In addition, h_1 and h_2 are the fabric apparent and stable thickness (under compressional pressure P), respectively.

Similarly, the recovery work WC' during the compressional recovery stage can be expressed by:

$$WC' = \int_{h_1}^{h_2} P'_h dh \tag{13}$$

where P'_h is the pressure load at the compression recovery stage at fabric thickness.

The compressional resilience coefficient (RC) is the ratio between recovery and compressional energy, which can be calculated as the follows:

$$RC = \frac{WC'}{WC} \times 100\% \tag{14}$$

The compressional linearity (LC) reflects the elasticity of a fabric against compression, which is reflected by

$$LC = \frac{WC}{\frac{1}{2}(h_1 - h_2)P} \tag{15}$$

Supplementary Note 8. Bilinear increasing behavior of electrical response with the compression force

We first explain the reason that the electrical output increases with the increasing of the compression force. In the open-circuit (OC) condition, when the external pressure is withdrawn, the V_{OC} will increase linearly corresponding to the vertical gap distance between the two layers (d) via the following equation:

$$V_{OC} = \frac{\sigma \cdot d}{\varepsilon_0} \quad (16)$$

where ε_0 is the permittivity in vacuum and σ is the triboelectric charge density.

In order to correlate the external pressure with the V_{OC}, the material resilience of TENGs was considered as the spring-entangled structure using the Hooker's law:

$$P = \frac{k \cdot x}{S} = \frac{k \cdot (d_0 - d)}{S} \quad (17)$$

where P is the pressure and k represents the elastic property of the material in TENGs. d_0 is the distance between two friction layers at the maximum separation state. Combining Supplementary Equation 16 and 17, we can get

$$\frac{V_{OC,0} - V_{OC}}{V_{OC,0}} = \frac{d_0 - d}{d_0} = \frac{S}{k \cdot d_0} \cdot P \quad (18)$$

Hence the relative variation of the V_{OC} should be expected to show a directly linear relationship with the applied pressure. Therefore, the voltage variation increases with the increasing of pressure.

The reason for different sensitivities can be attributed to the difference of the separation distance between two friction layers. In low pressure region, the increase of pressure results in drastic change of gap distance between two friction layers, as estimated in Supplementary Equation 17, which leads to larger voltage variations. When the pressure increases to a certain extent, the gap distance between two friction layers has already been close to 0. Therefore, further increasing the pressure will not change the separation distance, but only works for enhancing the effective contact area between the two friction layers. Therefore, the V_{OC} changes slowly and the sensitivity will be much smaller. In other words, the V_{OC} tends to saturate to zero, and the applied pressure has little effect on the voltage variation [11-12].

Supplementary Note 9. Experimental setup and conditions for vibrational energy harvesting

The vibration acceleration testing platform for vibrational energy harvesting is shown in Supplementary Fig. 21, which consists of a waveform controller (Tektronix AFG3102C) (Supplementary Fig. 21**b**), a power amplifier (YMC LA-800) (Supplementary Fig. 21**c**), and an electrodynamic shaker (Labworks ET-126 shaker) (Supplementary Fig. 21**d**). The electrical output was recorded by an electrometer (Keithley mode 6514). The electrodynamic shaker works as an external vibration source generating a harmonic vibration with controlled amplitude and frequency along the vertical direction. The vibration signal is generated from the waveform controller, amplified via the power amplified and finally utilized to control the vibration amplitude and frequency of the electrodynamic shaker. In this way, the acceleration and frequency of the resonator's movement can be accurately controlled. The vibrational frequency f can be set in the software. The vibrational acceleration α (m s^{-2}) can be determined by the following equation [13]:

$$V_{OC} = \frac{Q}{LC_0}\frac{1}{\omega^2}\alpha = \frac{1}{(2\pi)^2}\frac{\sigma S}{LC_0}\frac{1}{f^2}\alpha = k\frac{1}{f^2}\alpha \qquad (19)$$

where V_{OC} is the output voltage of the TENG, Q is the output charge, σ is the tribo-charge surface density, S is the activated area, k is the constant coefficient, ω is the forcing frequency, L is the length of the TENG, and C_0 is the permittivity of free space.

The proposed 3DB-TENG device is mounted on the acrylic bracket installed on a shaker. The specific test conditions of vibrational energy harvesting include the selected sinusoidal vibrational waveform, the vibrational frequency of 10 Hz, and the vibrational amplitude of 5 mm.

Supplementary Note 10. Experimental methods and conditions of one-month cycled test.

As for the method and times of the cycled measurement during one month, the details are listed as follows: a 3DB-TENG device is fixed on the optical platform, whose position remains unchanged during the whole month. The test period of each day is basically the same, and the continuous beating time per day is more than 3 hours in order to ensure the surface charge density has reached saturation. After each test, the test 3DB-TENG sample and the optical platform on which it is fixed are kept at constant temperature and humidity.

In addition, the applied conditions are consistent during one-month test, including the loading frequency of 3 Hz, the imposing force is 20 N, the contact and separation distance of 20 mm, and the activated area of $20\times20\times20$ mm^3. Moreover, all the tests are carried out under room temperature, normal humidity and pressure.

Therefore, we have tried our best to keep the experimental conditions as consistent as possible and keep the time of each day’s measurement over 3 hours.

Supplementary Note 11. The adopted washing process and condition for our 3DB-TENG

The experimental simulated washing environment was first cultivated in a beaker with household detergent and a magnetic stir bar added. Then, a 3DB-TENG device was directly put into the washing solution without any packaging. Afterward, the as-prepared beaker was placed on a magnetic stirrer to imitate agitator rotation in a household washing machine. The spinning speed of the magnetic stirrer was set at 600 rpm, the washing water temperature was kept around room temperature, and one washing cycle lasted for 20 min. Finally, the 3DB-TENG was rapidly dried in an oven at 60 ℃ for later electrical output measurement.

It is noteworthy that the simulated washing condition for our 3DB-TENG is also widely adopted by many other previous works [14-18], which is widely used to evaluate the washability of textile-based TENGs.

Supplementary Note 12. The analysis and processing method of the generated voltage signals

As shown in Supplementary Fig. 29, it can be found that the voltage signals collected by the multi-channel acquisition card are from the resistances which are connected with the sensing units (3DB-TENGs) in series. Therefore, the voltage signals are quite stable due to that the baselines of voltage signals are kept at 0 V, which enables us to compare the voltage signals from different sensing areas. In addition, since each step only corresponds to one black sensing block on the carpet, we just need to distinguish the most prominent voltage signal from the 64 channel signals to search the location of the stepping block. According to the fact that the voltage signal from the contact area is definitely larger than the interference signal from the non-contacted area, we can judge whether the sensing area is contacted or not by setting the threshold voltage. If it happens that more than one signal exceeds the threshold voltage, we can still lock the actual contacted area based on the maximum voltage signal. The flow diagram of algorithm of this process is illustrated in Supplementary Fig. 32.

Supplementary Note 13. The measures we took to reduce or avoid the potential unexpected conditions in the applications of self-powered identity identification carpet.

Appropriate Solutions if the participant steps on the junction of two black squares

We think that there are only three states if the participant steps on the junction of two black squares, i.e., stepping on no (Supplementary Fig. 33a), one (Supplementary Fig. 33b) or two sensing areas (Supplementary Fig. 33c).

However, the possibility of stepping on two sensing units at the same time can be excluded. As shown in Supplementary Fig. 33, our 3DB-TENG devices are sewn on the black squares in the shape of horseshoe (highlighted in blue). Taking the direction perpendicular to the door as an example, the longitudinal distance between two adjacent devices is about 30 cm, which is greater than the length of normal human feet (about 27-28 cm). Therefore, it is almost impossible to step on two sensing areas at the same time when walking towards the direction of the door. If the participants fail to follow the instruction (i.e., walking towards the direction of the door), it is also possible for the sole stepping on two sensing areas at the same time (Supplementary Fig. 33c). Under this circumstance, two voltage signal values will be generated. Based on the fact that the voltage signal generated in the main stepping area must be larger, we can easily lock the main/expected stepping area by comparing their voltage signal values.

In addition, stepping on no sensing areas means that most of the sole area falls on the white squares. This situation does exist in practical operation. However, we think that it can be avoided artificially when the participants know the operation specifications (e.g. only the black squares are valid) in advance.

Therefore, when operation specifications are followed, it is most likely that only one sensing area will be stepped on (i.e., only one voltage signal will be generated) if the participant steps onto the junction of two black squares. Even if operation specifications are not followed, the participant steps on no or two sensing areas. Stepping on no sensing area means wrong password and invalid authentication, which need to enter again. If stepping on two sensing areas, we can add data processing by distinguish the maximum voltage signal to lock the stepped area.

Therefore, there will be no trouble/confusion for the electric output collection and data recognition even if the participant steps onto the junction of two black squares.

Analysis of the hysteresis effect during signal acquisition or transmission

Although most of the data in our experiment are useful and present correct information, we cannot guarantee that there is no wrong information. It is undeniable that there are several unexpected situations in our experiment, which lead to wrong information, such as (1) electrode falls off accidentally, making it useless,

(2) stepping on the area without 3DB-TENG, leading to no signal generated, (3) stepping on two power generation areas at the same time, resulting in mixed signals, (4) hysteresis effect of signal transmission, as stated by the reviewer. In the following, we briefly analyze the possibility of these situations and the avoidance methods we took.

(1) We have fixed the connection position between yarn electrode and conductive wire by welding as well as wrapping with insulating tape, and checked repeatedly before the experiment. Therefore, the possibility of electrode falling off is small. (2) There is a possibility of stepping on the area without 3DB-TENGs. However, this issue can be avoided artificially when the entrant has already known the rules or matters needing attention that only stepping on the black blocks is valid. (3) Along the door direction, the length of normal foot (27-28 mm) is less than the length between two sensing areas (30 mm), so two sensing areas cannot be stepped on simultaneously. In addition, this situation can also be avoided artificially. (4) The data acquisition frequency of the multi-channel acquisition card is about 0.1 s, which is faster than the time required for a normal step (0.6-0.8 s). Therefore, under normal circumstances, it is almost impossible for hysteresis effect to lead to untimely data transmission. In addition, the update of hardware (e.g. acquisition card) and software (e.g. developed output program) can also effectively prevent the hysteresis effects.

Through these measures, we can effectively reduce the generation of false readings or wrong information.

Cross-talk analysis of the identity recognition carpet system

In fact, we have adopted many measures to reduce signal cross-talk between different sensing units. For example, (1) one signal unit only corresponds to one acquisition channel, (2) insulated wire is used as the connection medium, which can avoid the generation of electric signals of conductive wires, (3) the staggered and spaced design of black and white blocks can effectively prevent human feet from stepping on two sensing units at the same time. The position of sensing unit and its corresponding size are marked in Supplementary Fig. 33. As we know, the length of a normal foot is about 27-28 cm. if walking along the door direction, the nearest distance between adjacent sensing units is 30 mm, which is much larger than the length of human foot. Therefore, it is impossible to step on two sensing units at the same time when walking along the door direction. Of course, there is also the potential possibility of walking along other directions or stepping on the area without sensing units. However, these unexpected conditions can be avoided artificially according to relevant prompt information.

Therefore, we are very responsible to say that there is little or no signal cross-talk phenomenon during our test process. However, at present, we cannot guarantee that signal cross-talk does not exist at all or has been entirely eliminated. But this issue cannot eliminate the values of our new concept and initial application display. Moreover, we strongly believe that this issue can be easily solved through system optimization or

program improvement in future.

Analysis of potential password security

Here are some possible ways to eliminate or prevent potential peeping behaviors as much as possible.

1. Adding an isolation room where there is the identity recognition carpet, just like the arrangement of the bank's automatic teller machine (ATM). As shown in Supplementary Fig. 34a, when the visitor enters the front door (A), he/she can lock it back that can effectively prevent others from peeping.

2. Adding warning signs (Attention or no peeping) and information (e.g. "Make sure no one is watching you") at the entrance, as illustrated in Supplementary Fig. 34b. This is a protective measure based on the self-protection angle of the entrant.

3. We can modify the carpet structure and identification program according to the size of human foot, human weight, walking gait, and other indicators to associate the generated electrical signals with the unique walking states or behaviors of a specific person. These specific electrical signals, just like human fingerprints, are difficult or almost impossible to be replicated.

Moreover, we firmly believe that there will be more and better solutions to this problem with the development of technology.

Supplementary References

1. Niu, S. *et al*. Theoretical study of contact-mode triboelectric nanogenerators as an effective power source. *Energy Environ. Sci.*, **6**, 3576-3583 (2013).
2. Zi, Y., Niu, S., Wang, J., Wen, Z., Tang, W. & Wang, Z. L. Standards and figure-of-merits for quantifying the performance of triboelectric nanogenerators. *Nat. Commun.*, **6**, 1-8 (2015).
3. Chen, B. *et al*. Three-dimensional ultraflexible triboelectric nanogenerator made by 3D printing. *Nano Energy*, **45**, 380-389 (2018).
4. Xu, M. *et al*. High power density tower-like triboelectric nanogenerator for harvesting arbitrary directional water wave energy. *ACS Nano*, **13**, 1932-1939 (2019).
5. Jin, L. *et al*. Self-powered safety helmet based on hybridized nanogenerator for emergency. *ACS Nano*, **10**, 7874-7881 (2016).
6. Zhu, G. *et al*. Toward large-scale energy harvesting by a nanoparticle-enhanced triboelectric nanogenerator. *Nano Lett.*, **13**, 847-853 (2013).
7. Fan, X., Chen, J., Yang, J., Bai, P., Li, Z. & Wang, Z. L. Ultrathin, rollable, paper-based triboelectric nanogenerator for acoustic energy harvesting and self-powered sound recording. *ACS Nano*, **9**, 4236-4243 (2015).
8. Pan, L., *et al*. Liquid-FEP-based U-tube triboelectric nanogenerator for harvesting water-wave energy. *Nano Res.*, **11**, 4062-4073 (2018).
9. Zhang, B., *et al*. Rotating-disk-based hybridized electromagnetic-triboelectric nanogenerator for sustainably powering wireless traffic volume sensors. *ACS Nano*, **10**, 6241-6247 (2016).
10. Yao, B., Yan, L., Wang, J. & Hong, S. Test method for compression resilience evaluation of textiles. *Indonesian J. Elec. Eng. Comput. Sci*, **11**, 674-680 (2013).
11. Lin, L., *et al*. Triboelectric active sensor array for self-powered static and dynamic pressure detection and tactile imaging. *ACS Nano*, **7**, 8266-8274 (2013).
12. Zhu, G., *et al*. Self-powered, ultrasensitive, flexible tactile sensors based on contact electrification. *Nano Lett.*, **14**, 3208-3213 (2014).
13. Yu, H., *et al*. A self-powered dynamic displacement monitoring system based on triboelectric accelerometer. *Adv. Energy Mater.*, **7**, 1700565 (2017).
14. Gong, W., *et al*. Continuous and scalable manufacture of amphibious energy yarns and textiles. *Nat. Commun.*, **10**, 1-8 (2019).
15. Qiu, Q., *et al*. Highly flexible, breathable, tailorable and washable power generation fabrics for wearable electronics. *Nano Energy*, **58**, 750-758 (2019).
16. Dong, K., *et al*. A stretchable yarn embedded triboelectric nanogenerator as electronic skin for biomechanical energy harvesting and multifunctional pressure sensing. *Adv. Mater.*, **30**, 1804944 (2018).
17. Dong, K., *et al*. A highly stretchable and washable all-yarn-based self-charging knitting power textile composed of fiber triboelectric nanogenerators and supercapacitors. *ACS Nano*, **11**, 9490-9499 (2017).
18. Dong, K., *et al*. Versatile core-sheath yarn for sustainable biomechanical energy harvesting and real-time human-interactive sensing. *Adv. Energy Mater.*, **8**, 1801114 (2018).
