# Supplementary material for: Shape adaptable and highly resilient 3D braided triboelectric nanogenerators as e-textiles for power and sensing
Source: Nat Commun. 2020 Jun 8;11:2868. doi: 10.1038/s41467-020-16642-6 (PMC7280288; doi:10.1038/s41467-020-16642-6)
Supplement: Supplementary file 2 — Description of Additional Supplementary Files [file 41467_2020_16642_MOESM2_ESM.pdf]

Description of Additional Supplementary Files

File Name: Supplementary Movie 1
Description: Compression resilience of the 3DB-TENG.

File Name: Supplementary Movie 2
Description: 3DB-TENG lighting up hundreds of LEDs from simply tapping it by hand.

File Name: Supplementary Movie 3
Description: 3DB-TENG for powering low-power miniature wearable electronics.

File Name: Supplementary Movie 4
Description: An intelligent footwear system for motion monitoring and remote emergency rescue.

File Name: Supplementary Movie 5
Description: A self-powered identity recognition carpet for safeguarding entrances and early warning.
